# First adequately-known quadrupedal sirenian from Eurasia (Eocene, Bay of Biscay, Huesca, northeastern Spain)

**DOI:** 10.1038/s41598-018-23355-w

**Published:** 2018-03-23

**Authors:** Ester Díaz-Berenguer, Ainara Badiola, Miguel Moreno-Azanza, José Ignacio Canudo

**Affiliations:** 1 Grupo Aragosaurus-IUCA, Facultad de Ciencias, Universidad de Zaragoza, 50009 Zaragoza, Spain; 2 Dpto. Estratigrafía y Paleontología, Facultad de Ciencia y Tecnología, Universidad del País Vasco (UPV/EHU), Apartado 644, 48080 Bilbao, Spain; 3 Geobiotec, Dpto. de Ciências da Terra, Universidade Nova de Lisboa, 2829–526, Caparica, Portugal, Museo da Lourinhã, Lourinhã, Portugal

## Abstract

Sirenians are the only extant herbivorous mammals fully adapted to an aquatic lifestyle. They originated in Africa during the Paleocene from an undetermined clade of afrotherian mammals, and by the end of the Eocene they were widely distributed across the tropical latitudes. Here we introduce *Sobrarbesiren cardieli* gen. et sp. nov. It is the first adequately-known quadrupedal sirenian from Eurasia and the oldest record of this clade from western Europe. Fossils have been recovered from the middle Lutetian (SBZ15) site of Castejón de Sobrarbe-41 (Huesca, Spain), and comprise many cranial and postcranial remains, including pelvic girdle and hind limb bones, from at least six sirenian individuals of different ontogenetic stages. *Sobrarbesiren* shows a suite of characters previously considered synapomorphies of different clades of derived sirenians, such as the presence of the *processus retroversus* of the squamosal and the pterygoid fossa, combined with ancestral characters such as the presence of an alisphenoid canal, a permanent P5, at least two sacral vertebrae, a primitive pelvis and functional femora and fibulae. *Sobrarbesiren* is recovered as the sister taxon of Dugongidae and represents a transitional stage of adaptation to aquatic life between the amphibious quadrupedal prorastomids and the aquatic quadrupedal protosirenids.

## Introduction

Sirenians originated from an undetermined clade of afrotherian mammals in the Paleocene^1^. The fossil record of the group starts in the late early Eocene of the West Atlantic coast and Africa. The prorastomids *Prorastomus sirenoides*, from the late early Eocene or early middle Eocene^2^, and *Pezosiren portelli*, from the early middle Eocene^3^, both from Jamaica, are highly plesiomorphic forms and present a semiaquatic lifestyle^4^. Only fragmentary remains of prorastomids have been recovered from the late early and middle Eocene of the Old World^5,6^. Instead, the Eocene fossil record is mainly composed of more derived forms: the Protosirenidae (e.g. *Protosiren smithae*^7^, *Ashokia*^8^, *Libysiren*^9^), quadrupedal aquatic forms with reduction of the hind limbs^4^; and the Dugongidae (e.g. *Eotheroides*, *Eosiren*, *Halitherium*^10^), completely aquatic sirenians with an advanced stage of hind limb reduction.

All Eocene European sirenians are assigned to the Family Dugongidae^10^, with the exception of several fragmentary specimens of uncertain affinities^11,12^. Several dugongid species have been described in Europe: *Sirenavus hungaricus*^13^ and *Anisosiren pannonica*^14^ from the Lutetian of Hungary; “*Halitherium” taulannense* from the Priabonian of France^15^; and *Prototherium* spp., from the Bartonian of Spain^16,17^ and the Bartonian and Priabonian of Italy^18,19^. Until now, Eocene sirenian remains in the Pyrenean region had been scarce and fragmentary^20^. The new locality found in the central Pyrenees, the site of Castejón de Sobrarbe-41 (CS-41) in the province of Huesca (northeastern Spain), is an exceptional middle Lutetian sirenian bone bed, which has provided more than 300 remains from at least six sirenian individuals, of different ontogenetic stages, assigned to the new taxon. It is the oldest sirenian in western Europe and is represented by cranial and postcranial material including functional hind limbs, constituting the first adequately-known quadrupedal sirenian from Eurasia. The aim of this work is to describe this new sirenian, discuss its phylogenetic significance and study its adaptation to aquatic life.

### Geology and age

**Figure 1:**
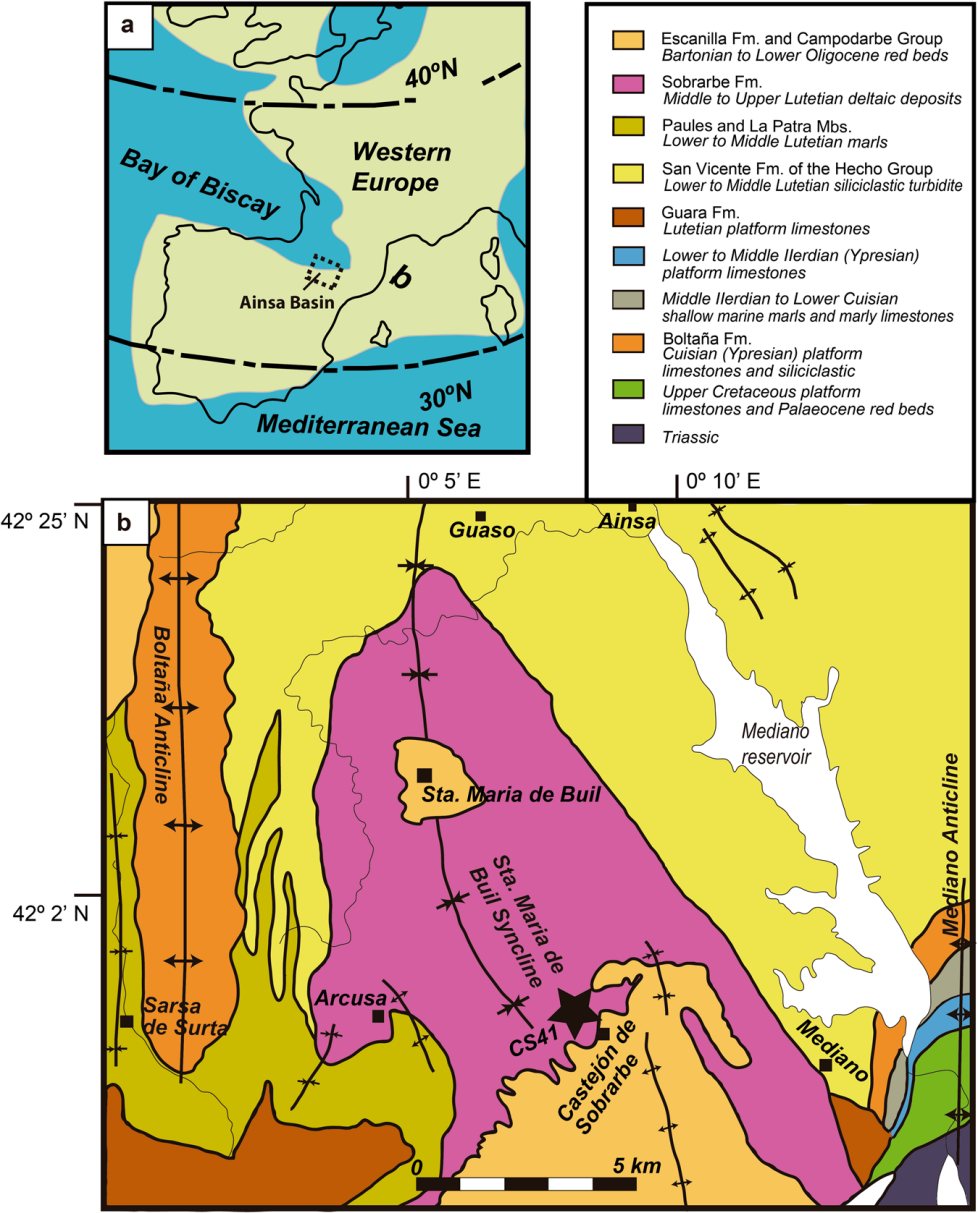
Geological location of the Castejón de Sobrarbe-41 fossil site. (**a**) Palaeogeography of NW Europe during the early Paleogene indicating the location of the studied area (blue areas: sea; green areas: land). Latitudes indicated are palaeolatitudes. Modified after Astibia, H. *et al*.^20^ New fossils of Sirenia from the middle Eocene of Navarre (Western Pyrenees): the oldest West European sea cow record, *Geological Magazine*, **147**, 665–673 (2010), reproduced with permission. (**b**) A simplified geological map of the studied area with the location of the middle Lutetian sirenian fossil site of Castejón de Sobrarbe-41 (CS-41), Huesca (Spain). Modified with permission from Mochales *et al*.^37^. Anisotropic magnetic susceptibility record of the kinematics of the Boltaña Anticline (Southern Pyrenees), Geological Journal, John Wiley and Sons. Software used: inkscape 0.91 https://inkscape.org/en/release/0.91/.

The Castejón de Sobrarbe-41 sirenian fossil site (CS-41) is located near the small village of Castejón de Sobrarbe (Comarca de Sobrarbe, Huesca, northeastern Spain). Geologically, this sirenian bone bed crops out in the Ainsa Basin (Fig. 1b), which constitutes the westernmost part of the Cenozoic Jaca-Pamplona Basin in the South Pyrenean Central Unit^21^. During the Eocene, this area was a deep marine gulf (the Bay of Biscay) located between the Iberian Peninsula and Europe (Fig. 1a), which opened northwestwards into the Atlantic Ocean, delimited to the east by the emergence of reliefs associated with the Pyrenean orogen^22^. The CS-41 fossiliferous level is in the uppermost part of the Sobrarbe Formation and has been correlated with nearby sections dated by magnetostratigraphy and benthic foraminifera^21^, placing CS-41 within the C19r chron and biozone SBZ15 (middle Lutetian, middle Eocene) and making these the oldest sirenian remains in western Europe. Over 300 disarticulated remains from at least six sirenian individuals, in different ontogenetic stages have been collected. The fossils are oriented but poorly sorted, with no signs of significant transport. This assemblage is interpreted as the infilling of an intertidal channel during a single energetic event, where all the remains dispersed over the tidal flat were trapped. Further data about the geological and taphonomic context of Castejón de Sobrarbe-41 can be found in the supporting information.

## Results

### Systematic palaeontology

Class Mammalia Linnaeus, 1758

Superorder Afrotheria Stanhope *et al*., 1998

Mirorder Tethytheria McKenna, 1975

Order Sirenia Illiger, 1811

Family uncertain

*Sobrarbesiren cardieli* gen. et sp. nov.

urn:lsid:zoobank.org:act:2AF65B38-E65D-429B-8946-C646FD48AE5E

urn:lsid:zoobank.org:act:960D79DB-2E74-4BA7-A7B1-13E8BFAD9B2C

#### Etymology

In reference to the Sobrarbe region (Huesca, northeastern Spain), and in honour of Jesús Cardiel Lalueza, who discovered the fossil site.

#### Holotype

**Figure 2:**
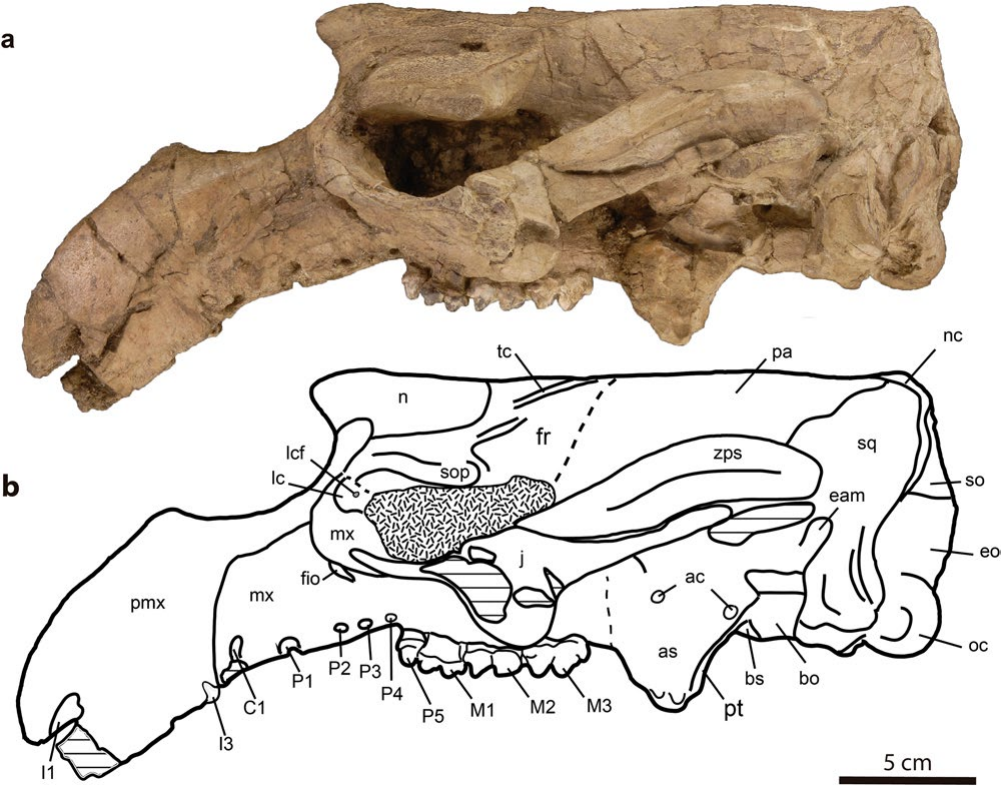
Skull of *Sobrarbesiren cardieli* gen. et sp. nov. (holotype, MPZ 2017/1), in lateral view. (**a**) Photograph, (**b**) interpretative sketch. Sutures are marked with plain lines; dashed lines represent reconstructed sutures; shaded areas represent broken or badly preserved bones; and dotted areas represent matrix. Anatomical abbreviations: ac, alisphenoid canal; as, alisphenoid; bo, basioccipital; bs, basisphenoid; eam, external auditory meatus; eo, exoccipital; fio, infraorbital foramen; fm, foramen magnum; fr, frontal; j, jugal; lc, lacrimal; lcf, lacrimal foramen; mf, mesorostral fossa; mx, maxilla; n, nasal; nc, nuchal crest; oc, occipital condyle; pa, parietal; pal, palatine; paop, paraoccipital process; pmx, premaxilla; pt, pterygoid; ptp, post-tympanic process; so, supraoccipital; sop, supraorbital process of frontal; sq, squamosal; tc, temporal crest; zps, zygomatic process of squamosal.

**Figure 3:**
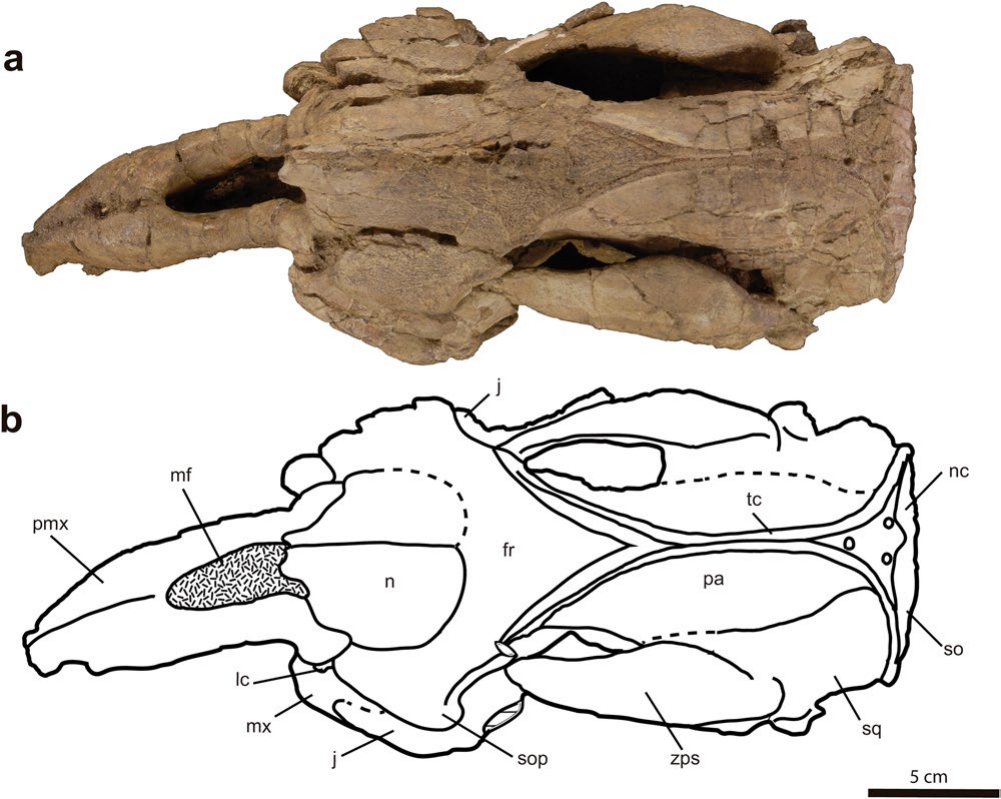
Skull of *Sobrarbesiren cardieli* gen. et sp. nov. (holotype, MPZ 2017/1), in dorsal view. (**a**) Photograph, (**b**) interpretative sketch. Sutures are marked with plain lines; dashed lines represent reconstructed sutures; and dotted areas represent matrix. For anatomical abbreviations see Fig. 2.

**Figure 4:**
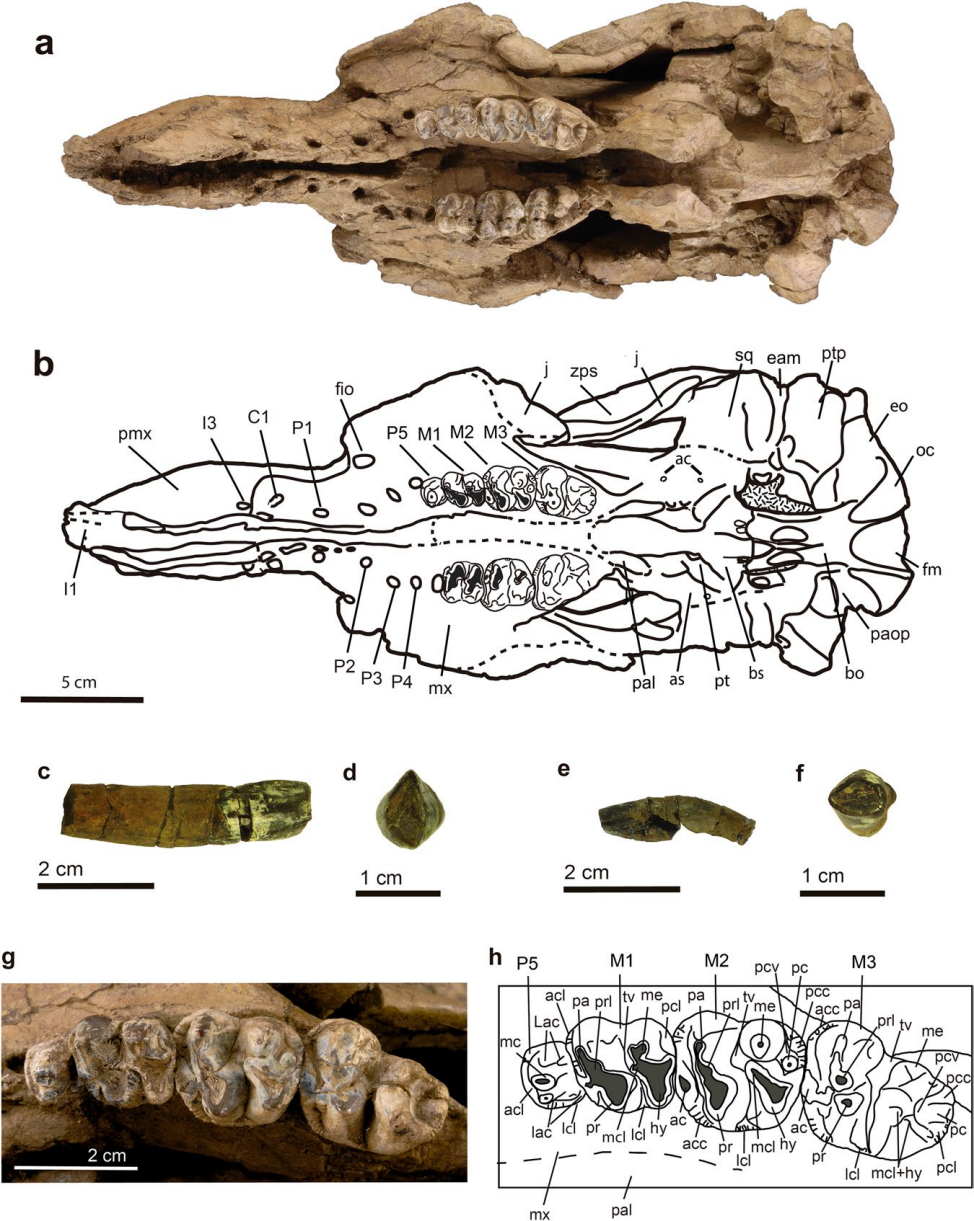
Skull of *Sobrarbesiren cardieli* gen. et sp. nov. (holotype, MPZ 2017/1), in ventral view and dentition (holotype, MPZ 2017/1 and MPZ 2017/4, MPZ 2017/5). (**a**,**b**) Skull (MPZ 2017/1) in ventral view. (**a**) Photograph, (**b**) interpretative sketch. For anatomical abbreviations see Fig. 2. (**c**,**d**) ?I1 (MPZ 2017/4) in lingual (**c**) and occlusal (**d**) views. (**e**,**f**) ?I3 (MPZ 2017/5) in lateral (**e**) and occlusal (**f**) views. (**g**,**h**) Left dental arcade of the holotype skull (MPZ 2017/1). (**g**) Photograph, (**h**) interpretative sketch. Sutures are marked with plain lines; dashed lines represent reconstructed sutures; and dotted areas represent matrix. Dental anatomical abbreviations: ac, anterior cingular cusp; acc, anterior accessory cusp; acl, anterior cingulum; hy, hypocone; Lac, labial cusp; lac, lingual cusp; lcl, lingual cingulum; mc, main cusp; mcl, metaconule; me, metacone; pa, paracone; pc, posterior cingular cusp; pcc, posterior accesory cusp; pcl, posterior cingulum; pcv, posterior cingular valley; pr, protocone; prl, protoconule; tv, transverse valley.

**Figure 5:**
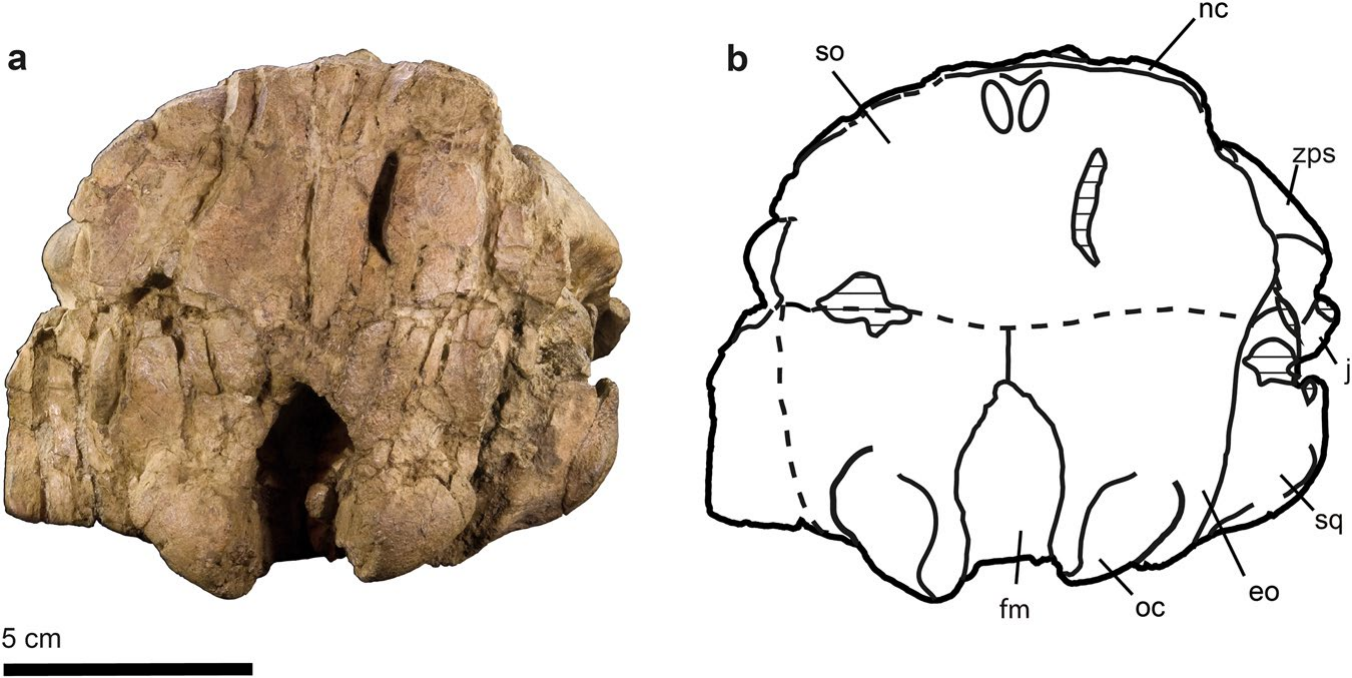
Skull of *Sobrarbesiren cardieli* gen. et sp. nov. (holotype, MPZ 2017/1), in posterior view. (**a**,**b**) Skull (MPZ 2017/1) in posterior view. (**a**) Photograph, (**b**) interpretative sketch. Sutures are marked with plain lines; dashed lines represent reconstructed sutures; and shaded areas represent broken or badly preserved bones. For anatomical abbreviations see Fig. 2.

MPZ 2017/1 a complete skull of a subadult individual which preserves alveoli of P1-4, a complete left P5, and M1-3 on both sides (Figs 2, 3, 4a,b,g,h and 5). The specimen is housed in the Museo de Ciencias Naturales de la Universidad de Zaragoza (MPZ) (Zaragoza, Spain). *S*. *cardieli* is the type species of the genus by monotypy and by original designation.

#### Paratypes

**Figure 6:**
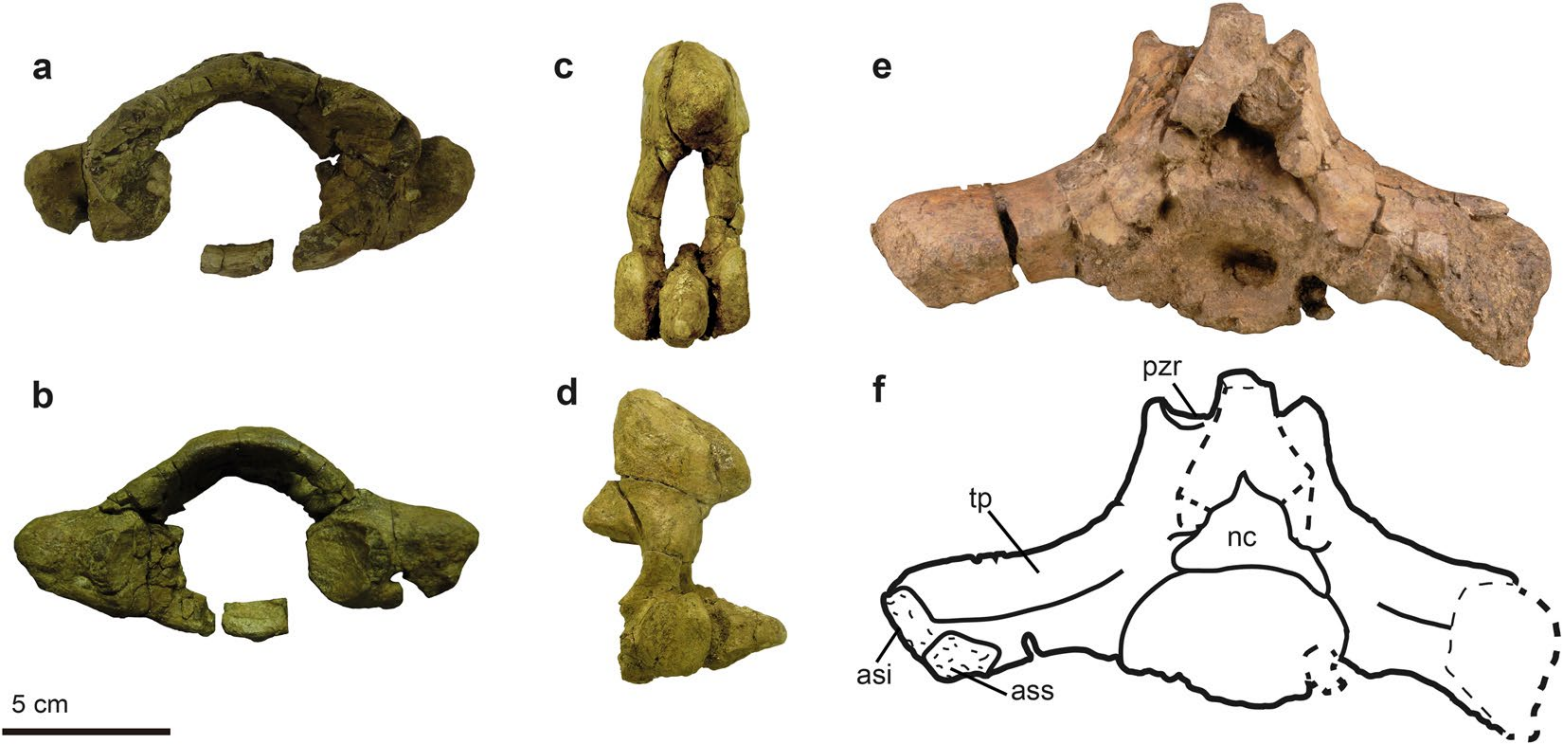
Vertebrae of *Sobrarbesiren cardieli* gen. et sp. nov. (**a,b**) Atlas (MPZ 2017/6) in anterior (**a**) and posterior (**b**) views. (**c**,**d**) Axis (MPZ 2017/7) in anterior (**c**) and lateral (**d**) views. (**e**,**f)** Sacral vertebra (MPZ 2017/18) in posterior view; (**e**) photograph, (**f**) interpretative sketch. Anatomical abbreviations: asi, articular surface for ilium; ass, articular surface for sacral vertebra; nc, neural canal; pzr, prezygapophyses; tp, transverse process. Dashed lines represent broken areas.

**Figure 7:**
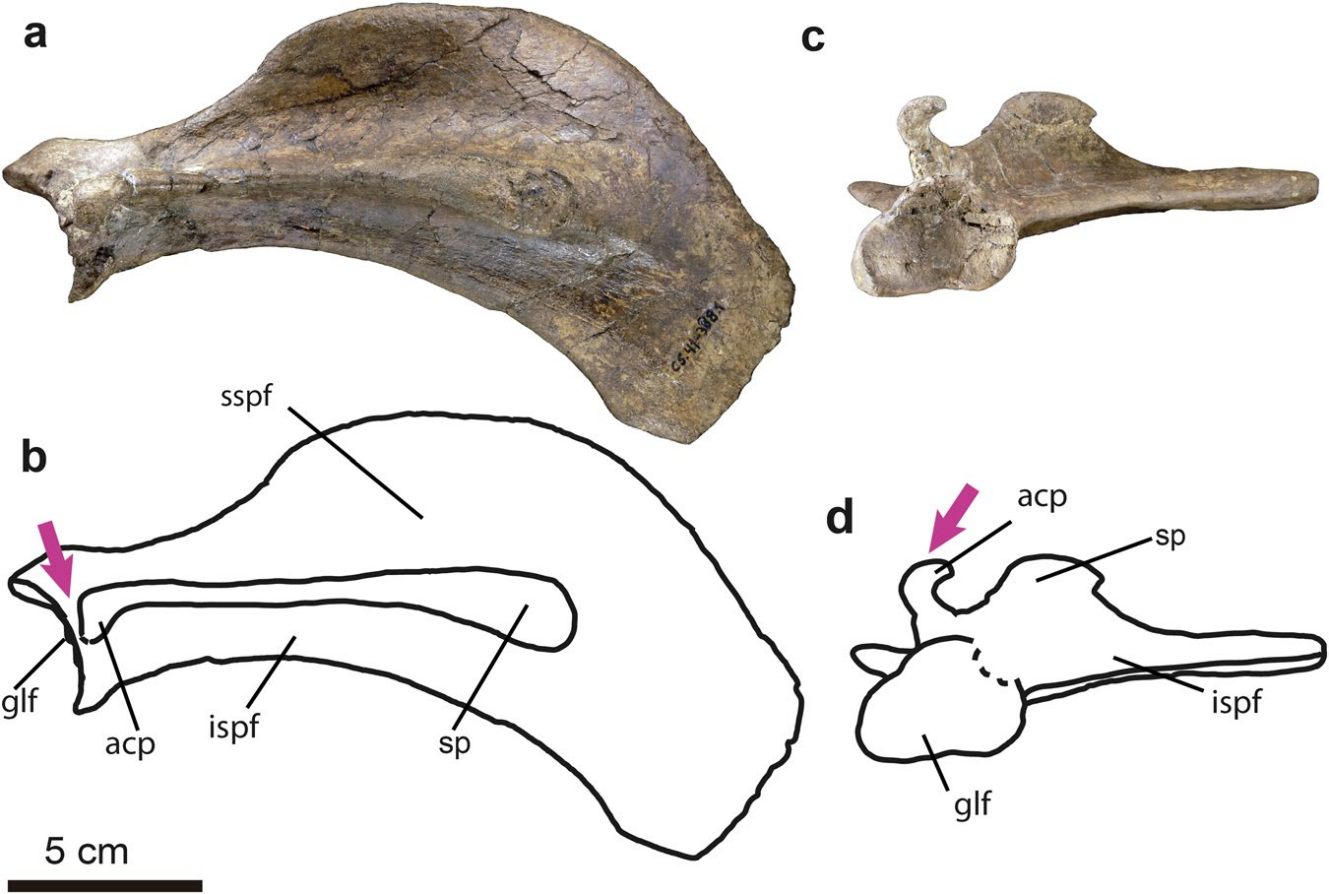
Scapula of *Sobrarbesiren cardieli* gen. et sp. nov. (**a**–**d**) Left scapula (MPZ 2017/40), in lateral (**a**,**b**) and ventral (**c**,**d**) views. (**a**–**c**) Photographs, (**b**–**d**) interpretative sketches. Anatomical abbreviations: acp, acromion process; glf, glenoid fossa; ispf, infraspinous fossa; sp, spine; sspf, supraspinous fossa. Dashed lines represent broken areas. Purple arrows mark autapomorphies discussed in the text.

**Figure 8:**
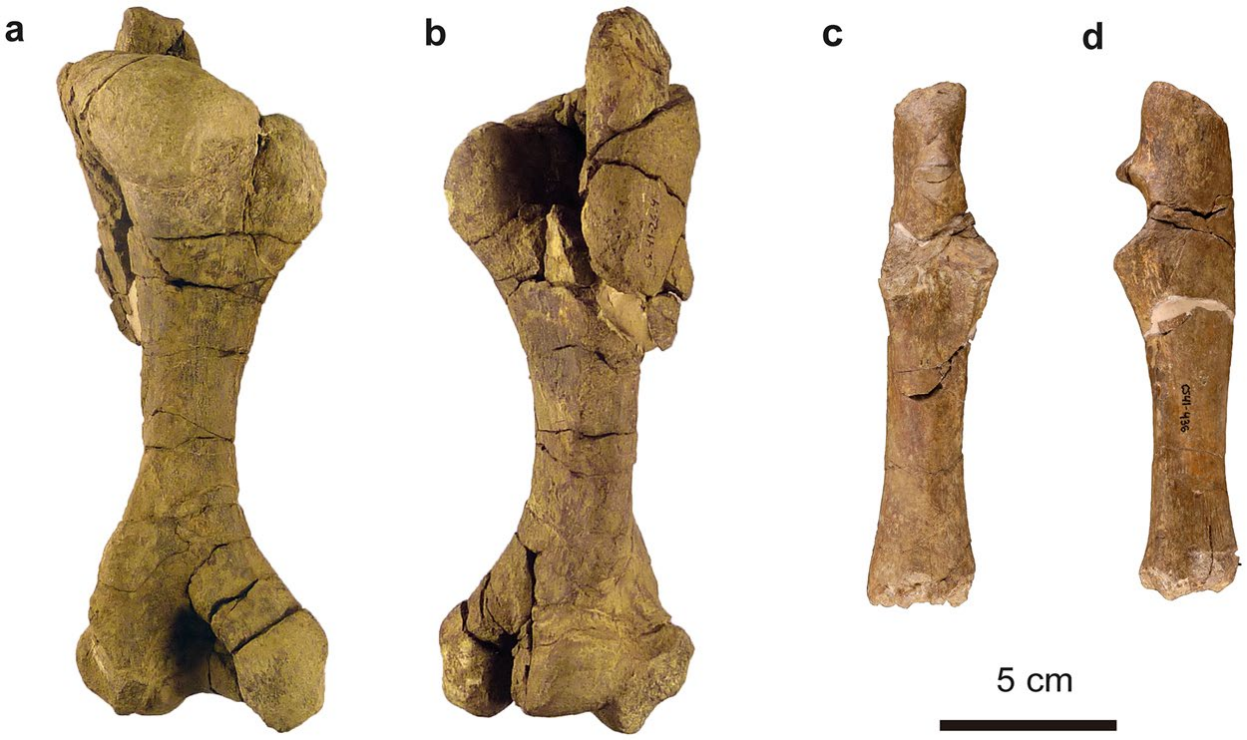
Forelimb bones of *Sobrarbesiren cardieli* gen. et sp. nov. (**a**,**b**) Left humerus (MPZ 2017/44) in anterior (**a**) and posterior (**b**) views. (**c**,**d**) Right ulna (MPZ 2017/46) in anterior (**c**) and medial (**d**) views.

**Figure 9:**
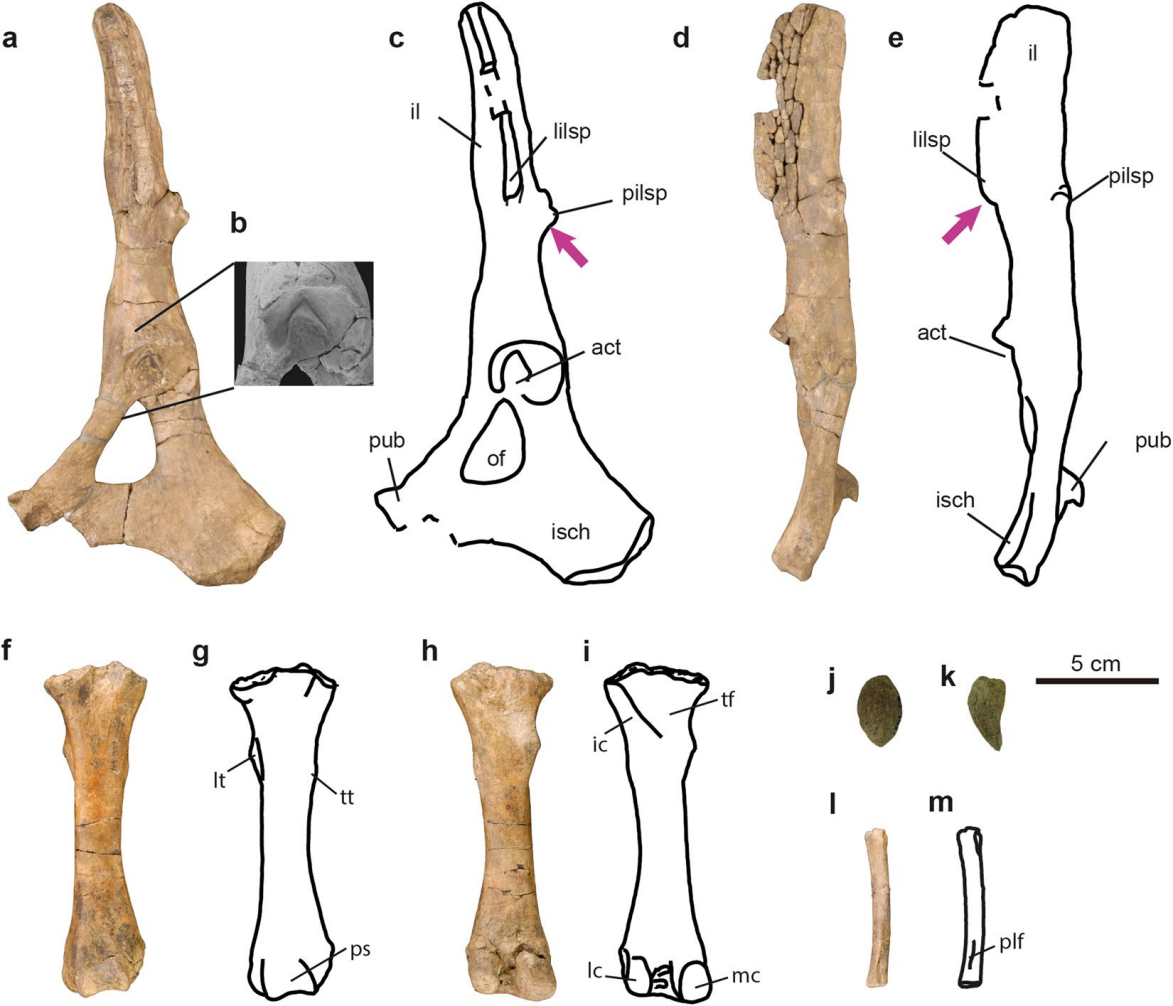
Innominate and hindlimb bones of *Sobrarbesiren cardieli* gen. et sp. nov. (**a**–**e**) Left innominate bone (MPZ 2017/47) in lateral (**a**–**c**) and dorsal (**d**,**e**) views. (**b**), detail of the acetabulum in lateral view. (**f**–**i**) Left femur (MPZ 2017/53) in anterior (**f**,**g**) and posterior (**h**,**i**) views. (**j**,**k**) Patella (MPZ 2017/52) in anterior (**j**) and lateral (**k**) views. (**l**,**m**) Left fibula (MPZ 2017/56) in posterior view. (**a**,**b**,**d**,**f**,**h**,**j**,**k** and **l**) Photographs, (**c**,**e**,**g**,**i** and **m**) interpretative sketches. Anatomical abbreviations: act, acetabulum; ic, intertrochanteric crest; il, ilium; isch, ischium; lc, lateral condyle; lilsp, lateral iliac spine; lt, lesser trochanter; mc, medial condyle; of, obturator foramen; pilsp, posterior iliac spine; plf, processus lateralis fibulae; ps, patellar surface; pub, pubis; tf, trochanteric fossa; tt, third trochanter. Dashed lines represent broken areas. Purple arrows mark autapomorphies discussed in the text.

Cranial and postcranial elements of at least six individuals, corresponding to different ontogenetic stages (Figs 6–9 and Supplementary Information Figs S2 and S3): MPZ 2017/2, an almost complete subadult skull that preserves the molar series on both sides, and lacks the exoccipital and basioccipital bones; MPZ 2017/3, premaxillary nasal process; MPZ 2017/4, ?first incisor; MPZ 2017/5, ?third incisor; MPZ 2017/6, atlas; MPZ 2017/7, axis; MPZ 2017/8 and MPZ 2017/9, bodies of cervical vertebrae; MPZ 2017/10 and MPZ 2017/11, anterior thoracic vertebrae; MPZ 2017/12, MPZ 2017/13, MPZ 2017/14 and MPZ 2017/15, thoracic vertebrae; MPZ 2017/16, anterior lumbar vertebra; MPZ 2017/17, posterior lumbar vertebra; MPZ 2017/18, sacral vertebra; MPZ 2017/19 and MPZ 2017/20, anterior caudal vertebrae; MPZ 2017/21, posterior caudal vertebrae; MPZ 2017/22, first left rib; MPZ 2017/23 - MPZ 2017/33, right ribs; MPZ 2017/34 - MPZ 2017/39, left ribs; MPZ 2017/40 - MPZ 2017/43, left scapulae; MPZ 2017/44, left humerus; MPZ 2017/45, right humerus, very distorted; MPZ 2017/46, right ulna that lacks the epiphyses; MPZ 2017/47, left innominate bone; MPZ 2017/48, incomplete right innominate bone; MPZ 2017/49 and MPZ 2017/50 juvenile right ischia; MPZ 2017/51 and MPZ 2017/52, patellae; MPZ 2017/53, left femur that lacks the proximal epiphysis; MPZ 2017/54, juvenile left femur; MPZ 2017/55 femoral head and MPZ 2017/56 left fibula.

### Locality and horizon

Only known from the Castejón de Sobrarbe-41 fossil site (CS-41), in Castejón de Sobrarbe, Huesca, Ainsa Basin in the southern Pyrenees, Sobrarbe Formation (middle Lutetian, early middle Eocene)^21^ (Fig. 1b).

### Diagnosis

(Ch. Character state following the descriptions and numbering sequence of Domning^23^ as updated by Vélez-Juarbe *et al*.^24^ and Springer *et al*.^1^ (e.g., Ch. 102 (0) refers to state 0 of character 102)): sirenian, based on the following synapomorphies: retracted and enlarged external nares (Ch. 8 (1)); premaxilla contacts the frontals (Ch. 9 (1)); and a P1-5, M1-3 postcanine dental formula (Ch. 155 (1)); and characterized by the following combination of characters (autapomorphies highlighted with*): upper dental formula 2.1.5.3 (I2 absent); pterygoid fossa present, extending above the level of the roof of the internal nares (Ch. 102 (1)); alisphenoid canal present (Ch. 101 (0)); rectangular and elongated basioccipital* (Fig. 4a,b and Supplementary Information Fig. S1a); hook-shaped acromion process of the scapula* (Fig. 7d), which extends just to the dorsal border of the glenoid fossa* (Fig. 7b); posterior iliac spine of the innominate bone present* (Fig. 9c); lateral iliac spine that appears abruptly on the lateral surface of the ilium with a flattened ventrolateral surface* (Fig. 9e).

### Differential diagnosis

*Sobrarbesiren* differs from “prorastomids”^2,3^ in having a deflected rostrum, a foramen ovale that is opened to form a notch (Ch. 103 (1)), a short sphenopalatine region, with a pterygoid process that is enlarged, thickened and downwardly projecting (Ch. 105 (1)), and in lacking the prominent sagittal crest seen in *Pezosiren*^3^ (Ch. 51 (1)).

It is distinguished from the Protosirenidae^7–9^ in having an enlarged rostrum (Ch. 3 (2)), a post-tympanic process of the squamosal that distinctly projects anteroventrally from the ventral squamosal border that intersects the paraoccipital process (Ch. 73 (0)), a cranial portion of the squamosal that reaches the parietal roof and the posterior part of the temporal crest (Ch. 76 (1)), a pterygoid fossa extending above the level of the roof of the internal nares (Ch. 102 (1)) (this character is probably absent in *Ashokia antiqua*^8,9^), and it differs from all protosirenids except *Ashokia antiqua* in having an outline of the zygomatic process of the squamosal that is gradually tapered and pointed anteriorly (Ch. 81 (0)). *Sobrarbesiren* shares with *Ashokia antiqua* and *Libysiren sickenbergi* long nasals that are slightly separated by frontals posteriorly^8,9^.

*Sobrarbesiren* is distinguished from the Dugongidae in having an alisphenoid canal (Ch. 101 (0)), a permanent fifth premolar present (Ch. 146 (0)), a small infraorbital foramen (Ch. 13 (0)) only shared with *Eotheroides aegyptiacum*, and in having well-developed (i.e. non-vestigial) innominate bones, presenting a large obturator foramen and a deep acetabulum, and complete hind limbs still present.

*Sobrarbesiren* is further differentiated from *Sirenavus hungaricus*^12,13^ in having a zygomatic-orbital bridge of the maxilla elevated more than 1 cm above the alveolar margin (Ch. 11 (1)); a post-tympanic process of the squamosal present, and M3 larger than the other upper molars. *Sobrarbesiren* differs from the genus *Eotheroides* in that the latter has a longer median process of the frontals, resulting in a bigger notch between the nasals. On the other hand, both taxa share swollen and dense (pachyosteosclerotic) anterior ribs that are banana-shaped. *Sobrarbesiren* is distinguished from *Eosiren* in having a premaxillary symphysis that extends less than one-third of the skull length, large nasal bones, and swollen anterior ribs. *Sobrarbesiren* differs from *Prototherium* in not showing dolichocephaly (see Supplementary Information Table S1), in having a lesser degree of rostral deflection (see table 1 in^17^) and in having an enlarged rostrum (Ch. 3 (2)) (except *Prototherium intermedium)*. It is distinguished from “*Halitherium” taulannense* in having I3 present, a zygomatic-orbital bridge of the maxilla elevated more than 1 cm above the alveolar margin (Ch. 11 (1)) and an external auditory meatus that is narrow and slit-like (Ch. 82 (0)).

### Description

**Figure 10:**
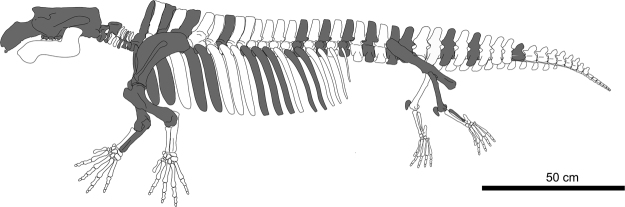
Reconstructed composite skeleton of *Sobrarbesiren cardieli* gen. et sp. nov. Shaded elements represent the fossils studied.

*Sobrarbesiren* is a medium-sized quadrupedal sirenian with an estimated total length of 2.7 m (Fig. 10). The skull descriptions are based on the holotype (MPZ 2017/1) and the paratype (MPZ 2017/2).

The holotype skull shows an open basioccipital-basisphenoid suture and a M3 with minor wear, suggesting that the specimen represents a subadult individual^24^ (Fig. 4a,b,g,h). The holotype skull shows a 32° rostral deflection, which is greater than in prorastomids^2,3^ but less than in more derived sirenians^7,17^. The premaxillary symphysis is enlarged (Ch. 3 (2)) and laterally compressed (Ch. 10 (1)). The anteroventralmost end of the suture with the maxilla is on the vertical level of the symphyseal summit. The nasal process of premaxilla is thin and tapering at the posterior end (Ch. 6 (0)). It contacts ventrally the maxilla and posteriorly the nasal, and as in most other sirenians, it contacts the frontals^23^. The external nares are retracted and enlarged to the level of the anterior margin of the orbit (Ch. 8 (1)). The nasals are large and meet along their dorsomedial borders (Ch. 31 (0)) (internasal suture = 65 mm), as in prorastomids^2,3^, some protosirenids^8,9^ and Eocene dugongids^15,17,25^. The anterior borders of the nasals together form a v-shaped extension beyond the mesorostral´s fossa posterior edge **(**Ch. 37 (0)). The posterior edges of the nasals are rounded, and they are shallowly separated by the frontals (Fig. 3) in a way similar to *Ashokia antiqua*^8^. The lacrimals are partially preserved. The anterior border of each is rounded, and the posterior border is broken. They are surrounded by the maxilla and the frontal (Ch. 93 (0)). A nasolacrimal foramen is present (Ch. 91 (0)). The frontal roof is flat (Ch. 42 (0)) and does not bear knoblike bosses (Ch. 45 (0)). The supraorbital processes of the frontals are stout and dorsoventrally flattened and show a prominent dorsolateral corner (Ch. 36 (0)). Their lateral borders are not divided (Ch. 44 (0)). The lamina orbitalis of the frontal is covered by sediment (Ch. 38 (?)). The elongated and convex parietals show smooth temporal crests that join just in front of the coronal suture for a total of 52.5 mm in craniocaudal length (Fig. 3). A true sagittal crest is absent (Ch. 51 (1)), as in all other sirenians^23^ except *Pezosiren*^3^, because the fused temporal crests are not raised. In the paratype skull (MPZ 2017/2) the temporal crests are separated by 3 mm. The supraoccipital shows a pronounced nuchal crest positioned at the same level as the skull roof, but it is less massive than in protosirenids^7^. The morphology of the supraoccipital is unclear (Fig. 5a,b, Ch. 64 (?)). Three emissary foramina forming a triangle are situated on the skull roof just anterior to the nuchal crest (Fig. 3). The exoccipitals meet along a suture dorsal to the foramen magnum (Ch. 66 (0)). Their dorsolateral borders are rounded and smooth (Ch. 70 (1)). The hypoglossal foramen is not discernible (Ch. 72 (?)). The basioccipital is elongated and rectangular in ventral view. It measures 37 mm along its ventral length (from the line of fusion with the basisphenoid to the base of the foramen magnum) and 17.5 mm across its body. Its ventral surface shows a median keel that bifurcates into the basisphenoid (Fig. 4a,b). It differs from the basioccipital of prorastomids, which is transversely wider posteriorly than anteriorly^2^ (Supplementary Information Fig. S1b), and from those of protosirenids and dugongids, which show a waisted body (Supplementary Information Fig. S1,c,d). An alisphenoid canal is present (Ch. 101 (0)), as in prorastomids^2,3^ and protosirenids^7,26^. The foramen ovale is converted into an open notch (Ch. 103 (1)). The pterygoid processes are enlarged (Ch. 105 (1)), as in all sirenians except prorastomids^3^. They project ventrad, showing a shallow pterygoid fossa (Ch. 102 (1)) that is absent in protosirenids^7–9^. The palatines are <1 cm thick at the level of the penultimate cheek tooth (Ch. 16 (0)). Their anterior border extends anteriorly beyond the posterior edge of the zygomatic-orbital bridge (Ch. 99 (0)) (Supplementary Information Fig. S2c). Their posterior border is broken (Ch. 97 (?)). The zygomatic-orbital bridge of the maxilla is long anteroposteriorly (Ch. 14 (0)). Its anterior edge is thin and sharp, and the posterior one is thicker and rounded (Ch. 22 (0)). It is elevated more than 1 cm above the alveolar margin (Ch. 11 (1)), as in prorastomids^2^ and the protosirenid *Ashokia antiqua*^8^. There is a narrow palatal gutter (Ch. 23 (0)) along the midline of the maxillae, as in dugongids but not in protosirenids^1^. The infraorbital foramen is small (Ch. 13 (0)) (height: 16 mm; width: 8.3 mm) as in basal sirenians^24^, and unobstructed (Ch. 20 (0)). The squamosal reaches the skull roof and the temporal/nuchal crest (Ch. 76 (1)) (Fig. 2). The zygomatic process of the squamosal is tapered anteriorly (Ch. 81 (0)). Its medial side is concave and inclined inward dorsally (Ch. 84 (0)). The sigmoidal ridge is present, and it is prominent (Ch. 74 (2)). The *processus retroversus* is present, as in all dugongids^23^ and *Libysiren sickenbergi*^9^, and is moderately inflected (Ch. 77 (1)). The external auditory meatus is narrow (Ch. 82 (0)), as in most basal sirenians, and it is higher dorsoventrally than anteroposteriorly, unlike in the prorastomid *Pezosiren*^3^. The post-tympanic process is enlarged and projects anteroventrally, bearing a facet for insertion of the sternomastoid muscle (Ch. 73 (0)). The preorbital process of the jugal is a thin plate (Ch. 88 (0)) that contacts the maxilla (Ch. 87 (0)). The ventral process of the jugal is broken and shifted (Ch. 85 (?)) and the zygomatic process is longer than the anteroposterior diameter of the orbit (Ch. 89 (0)).

The upper dental formula is 2.1.5.3 (Ch. 145 (0); Ch. 146 (0); Ch. 150 (0); Ch. 155 (1)). There is a broken alveolus for the tusk at the tip of each premaxilla (Ch. 139 (0)) (Fig. 4a,b), which are shorter than half of the premaxillary symphysis length (Ch. 140 (0)). There is no sign of I2. The left alveolus situated at the posterior end of the premaxilla, just before the premaxilla-maxilla suture, is thus assigned to I3 (Ch. 143 (0)) (Fig. 4a,b and Supplementary Information Fig. S2c). The absence of I2 is a derived condition with respect to the 3.1.5.3 dental formula of other basal sirenians^27^. Two isolated teeth are tentatively identified as a ?I1 and a ?I3 (Fig. 4c–f). The tusk is lens-shaped (Ch. 141 (1)) with enamel on all sides of the crown (Ch. 142 (0)), which is differentiated from the root (Ch. 137 (0)). The cross section of the ?I3 crown is suboval and its length (6,5 mm) is considerably smaller than that of ?I1 (15 mm). Alveoli of canines and premolars in both skulls indicate that these teeth were single-rooted (Ch. 144 (1) and (Ch. 157 (1)) (Fig. 4a,b and Supplementary Information Fig. S2c). The left P5 is preserved in the holotype, which is a plesiomorphic state shared with prorastomids and protosirenids^2,3,7^. The P5 is a tiny tooth in comparison with molars, with a main central cusp surrounded by much lower lingual and labial accessory cuspules. There is an anterior cingulum with small cingular cuspules too. The length of each molar is greater than its width, with this condition being more pronounced on M2-3 (see Supplementary Information Table S2). The three teeth are bilophodont three-rooted molars, with trigon higher than talon, and thus with protoloph (protocone, protoconule and paracone) higher than the paraloph (hypocone, metaconule and metacone) (Fig. 4g,h). Both lophs are quite worn in the M1-2 but well developed protoconule and metaconule are present in the three molars. The lophs are separated by a deep transverse valley, open labially but closed lingually by a cingulum; in the M1-2 it is closed in its half way through due to the presence of the highly worn metaconule. The labial and lingual cusps seem not to be transversally aligned along the labial-lingual axis but slightly oblique. There are anterior and posterior cingulae present, which are more prominent distally in the dental arcade, and several anterior accessory cusps located mesial to paracone and protocone, and with a large posterior accessory cusp distal to metacone in the M3. In the M2-3 the posterior cingular valley is present, though it is incipient in the M2.

The full vertebral formula is unknown (Ch. 200 (?) and Ch. 204 (?)). The vertebrae lack the horizontally projecting flanges at the tip of the neural spine described in prorastomids^3,5^ or the bifurcated neural spines shown by protosirenids^7^ (Fig. 6d and Supplementary Information Fig. S3a). The atlas is wider than high (Supplementary Information Table S3) with a low dorsal arch, and has large posterior cotyles as in prorastomids^2^, but the transverse processes are rounded and knob-like, like those of basal dugongids^25^ (Fig. 6a,b). The axis has a robust and swollen neural spine strongly inclined cranially (Fig. 6c,d). The odontoid process is as long as the vertebral centrum. The centra of the cervical vertebrae are compressed craniocaudally. The anterior thoracic vertebrae have high neural spines like prorastomids^3^ and protosirenids^7^, but teardrop-shaped neural canals, as in dugongids^16,25^ (Supplementary Information, Fig. S3a).

*Sobrarbesiren* has a sacrum that comprises at least two unfused sacral vertebrae (Ch. 205 (1)), by contrast with protosirenids and dugongids, which are characterized by a single sacral vertebra^4^. The transverse processes are short (Fig. 6e) compared to non-prorastomid sirenians^7,25^. The articular surfaces for the ilium are diamond-shaped, short anteroposteriorly and dorsoventrally expanded (47.5 mm in dorsoventral height), unlike the large articular surfaces of *Pezosiren portelli*^3^. The posterior border of the transverse process is flattened and shows a bevelled rhomboidal articular surface for a more posterior sacral vertebra that is still unknown (Fig. 6e,f). The caudal vertebrae have dorsoventrally flattened transverse processes and chevron facets. The presence/absence of a fluke cannot be determined based on the available specimens (Ch. 207 (?)).

The ribs are pachyosteosclerotic. The first rib has a broadened, truncated and flattened distal end (Supplementary Information Fig. S3b) as in some dugongids^16,25^. On the ventral side of its neck, there is no process for origin of the longus capitis muscle. The succeeding anterior ribs are banana-like (Supplementary Information Fig. S3c, see also Supplementary Information Table S4 for rib measurements), similar to the dugongid *Eotheroides*^25^. The posterior ribs are slender, lacking the swollen diaphysis of the anterior ribs. The smooth articular surfaces of the rib heads indicate a synovial articulation unlike in protosirenids^28^.

The scapula is sickle-shaped and stout (see Supplementary Information Table S5 for measurements), as in Eocene dugongids^16,25^. The acromion process of the scapula is hook-shaped, protruding laterodistally and turning posteriorly, showing a well-marked anterior angle (Fig. 7a,b), and the acromion extends just to the dorsal border of the glenoid fossa, features not observed in any other sirenian. The acromion process is very fragile and is not preserved in many of the Eocene taxa. Nevertheless, the morphology and extension of the acromion process of the left scapulae MPZ 2017/40 (Fig. 7) and MPZ 2017/42 of *Sobrarbesiren* are different from any other known sirenian. In the holotype right scapula of *Protosiren smithae* (CGM 42292, Cairo Geological Museum (Egypt); cast, USNM 94810, US National Museum of Natural History), the distance between the edge of the acromial process and the dorsal border of the glenoid cavity is 15 mm and the acromion is more robust and massive, without such a marked turn at the distal end. *Eosiren libyca* also has a massive acromial process that ends before reaching the dorsal border of the glenoid cavity (see^29^, plate III, figure 1a,b). “*Halitherium” taulannense* shows a distance of 30 mm between the acromion and the glenoid cavity. The distal edge of the process is not complete in this taxon. *Eotheroides sandersi* shows an acromial process that continues, without turning, in the direction of the spine axis until its end, and does not reach the glenoid cavity (see^25^, figure 59, A).

The humerus is robust (Ch. 221 (1)), with strongly developed proximal and distal epiphyses that are similar in width (Fig. 8a,b, and Supplementary Information Table S6), as in protosirenids^7^ and Egyptian Eocene dugongids^25^, but unlike the humerus of *Pezosiren*, which shows a proportionally broader distal epiphysis^3^. The bicipital groove is wide (Ch. 213 (0)) (see Supplementary Information Table S6 for measurements), and the olecranon and coronoid fossae are distinct and deep.

The ulna has a straight shaft and is not fused to the radius, but this is probably due to inmaturity of the specimen (Fig. 8c,d). The olecranon is long (30 mm), as in *Protosiren smithae* (see^7^, figure 10). It is straight and coaxially aligned with the main axis of the shaft, and its cranial side is convex unlike in other sirenians, where it slopes backward^7,25^.

The innominate is long and narrow with a long pubic symphysis (Ch. 215 (0)) (Fig. 9a,c). The rod-like ilium resembles that of protosirenids^30^, but the expanded ischium is directed posterolaterally and is less curved. The deep acetabulum (Fig. 9b, see also Supplementary Information Table S7 for measurements), the well-developed, teardrop-shaped obturator foramen, and the distance between this foramen and the beginning of the sacroiliac joint, resemble the conditions in *Pezosiren*^3^. The innominate of *Sobrarbesiren* differs from those of all other sirenians in having a posterior iliac spine, and a lateral iliac spine that arises abruptly on the lateral surface of the ilium (Fig. 9c–e). This lateral spine shows a flattened ventrolateral surface (Fig. 9a,c). The patella (Fig. 9j,k) is similar to the teardrop-shaped patella of *Pezosiren*^3^. The femur lacks the proximal epiphysis. This bone resembles that of *Pezosiren*^3^ and protosirenids^7^ in having a deep intertrochanteric fossa, and a distal epiphysis with robust condyles (Fig. 9f–i). *Sobrarbesiren* differs from *Pezosiren*^3^ in having a reduced third trochanter, which is absent in protosirenids^7^. The fibula of *Sobrarbesiren* is the oldest sirenian fibula known. It is slender and shows a triangular distal articular surface, whereas this is rounded in the holotype fibula of *Protosiren smithae* (CGM 42292; cast, USNM 94810). A distolateral crest is present (Fig. 9l,m), though absent in USNM 94810.

## Discussion

### Phylogenetic analysis

#### Results

**Figure 11:**
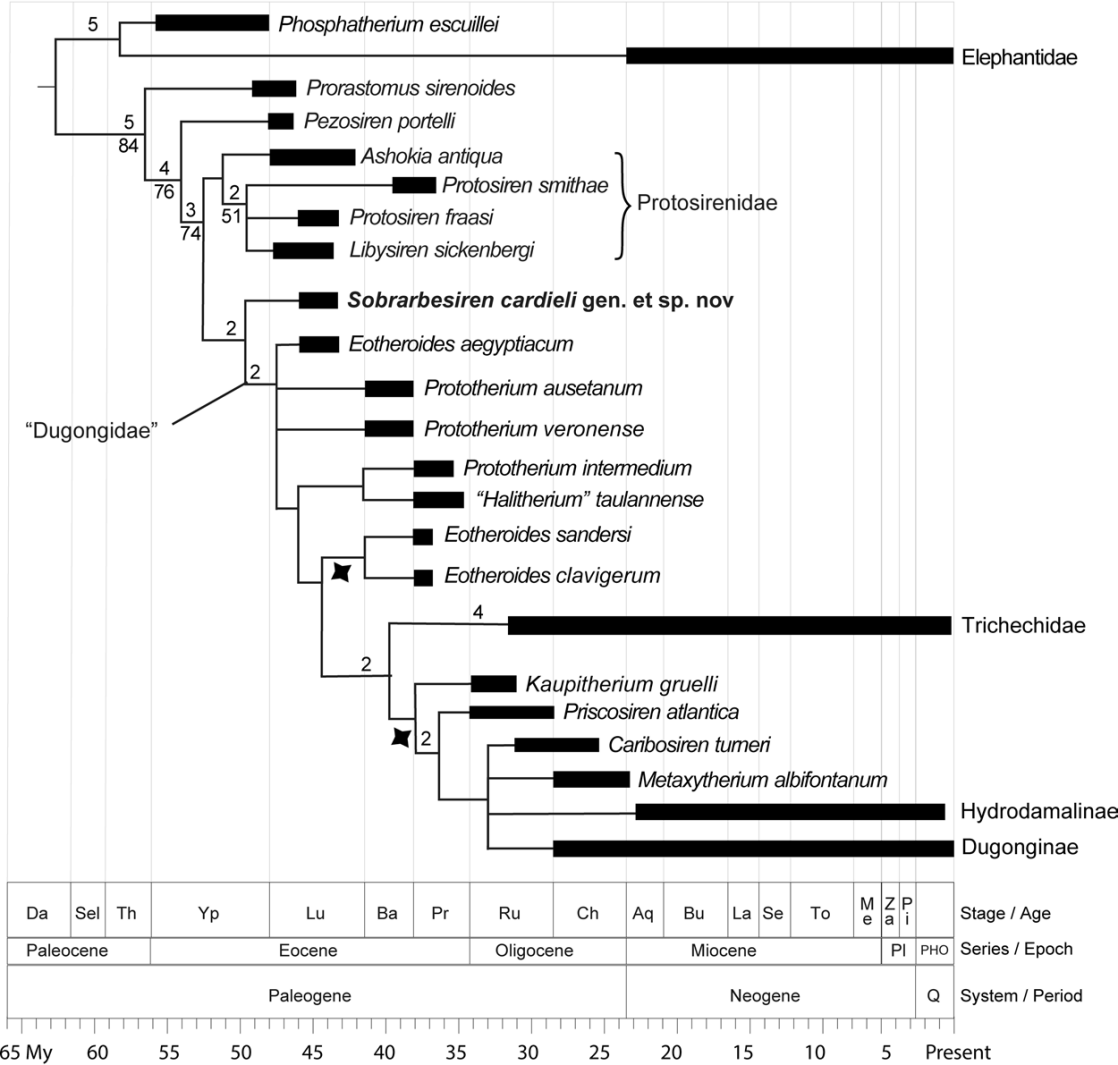
Phylogenetic relationships of *Sobrarbesiren cardieli* gen. et sp. nov. among the main sirenian clades. Time-calibrated reduced strict consensus of 96 most-parsimonious trees of 268 steps. *Sobrarbesiren* is recovered as the sister taxon of the clade “Dugongidae”. Note that our results do not support a strictly monophyletic Dugongidae, as Trichechidae nest inside this family. Numbers over branches represent Bremer support values over 1. Numbers below branches represent Bootstrap values over 50. This topology is obtained after *a posteriori* pruning of *Eotheroides lambondrano*, recovered as a wild card taxon. Stars below branches show the possible location of this taxon.

The analysis resulted in 96 most-parsimonious trees of 268 steps (Consistency Index, CI 0.433; Retention Index, RI 0.776, Rescaled Consistency Index, RC 0.336). *Sobrarbesiren* is recovered at the base of the clade that includes all sirenians except *Prorastomus sirenoides*, *Pezosiren portelli* and the clade Protosirenidae, as the sister taxon of all dugongids and trichechids (Fig. 11). The general topology of the tree resembles that of other authors^1^, but important differences arise. First, Protosirenidae is recovered as a monophyletic group, formed by the genera *Protosiren*, *Libysiren* and *Ashokia*, although with low support. Second, Trichechidae is returned to a more derived position, nesting within Dugongidae, as the sister group of the most exclusive clade that includes *Kaupitherium* (=*Halitherium schinzii* of other authors), Dugonginae and Hydrodamalinae. Another notable result of our analysis is that the genera *Eotheroides* and *Prototherium* are not recovered as monophyletic.

*Sobrarbesiren cardieli* is recovered as the sister taxon of Dugongidae. We refrain from including it within this family for two reasons: first, the current diagnosis of Dugongidae is synapomorphy-based and requires the members of the clade to lack the P5, the alisphenoid canal, and well-developed hind limbs^1,23^, a diagnosis not shared by *Sobrarbesiren*. Second, we consider that the current topology of the tree may change in the future, as a deeper split between Dugongidae and Trichechidae is plausible considering the available data. In this scenario, *Sobrarbesiren* will probably remain stable as a stem sirenian.

### Lifestyle

*Sobrarbesiren* shows several characters proving that it retained some capacity for supporting its own weight on land. The anterior thoracic vertebrae have tall neural spines, indicating the presence of a nuchal ligament that could support the head^3,7^. The long and rugose pubic symphysis of the innominate bone shows that there is a bony connection between the two pubes. This feature, together with the presence of a bony sacroiliac joint, is a prerequisite for supporting the body weight on land^30^, in contrast to the weak sacroiliac joint of protosirenids, which probably ruled out quadrupedal locomotion on land in favour of a pinniped-like motion^27^.

The presence of an unfused sacrum composed of at least two sacral vertebrae is a plesiomorphic state compared with the sacra of protosirenids and dugongids, which are composed of only one vertebra^4^. Nevertheless, the elongated and anteroposteriorly shortened transverse processes of the sacral vertebrae of *Sobrarbesiren* suggest an adaptation to an aquatic lifestyle, in contrast to the short transverse processes with anteroposteriorly expanded articular surfaces of *Pezosiren*, which are similar to those of land mammals^3^.

The large femoral acetabulum of the innominate bone shows a well-marked attachment area for the round ligament (Fig. 9b), indicating that the femoral head was strongly anchored in the acetabulum. This implies an active role of the femur in either walking or swimming (or both), which contrasts with the evidently vestigial function of this bone in more derived sirenians^4^. The distal crest observed on the fibula of *Sobrarbesiren* is probably the *processus lateralis fibulae* described in the quadrupedal proboscidean *Numidotherium koholense*^31^, to which the peroneal muscles were presumably attached. This may well be a plesiomorphic feature, and it is absent in the fibula cast (USNM 94810) of the protosirenid *Protosiren smithae*, the only other sirenian fibula known. Even though no autopodial elements from the hind limb have been identified, the presence of articulation structures of the knee such as the patellar articular surface of the femur (Fig. 9g), the preserved patella, and a well-formed fibula supports the presence of a complete hind limb with a mobile foot^7^.

*Sobrarbesiren* shows further adaptations to life in water, such as pachyosteosclerotic bones, an enlarged rostrum and retracted nares, together with an unfused sacrum, a rod-like ilium and relatively short femur and fibula. This combination of aquatic and terrestrial adaptations suggests an amphibious lifestyle, whereby *Sobrarbesiren* spent most of its time feeding in shallow waters but was capable of moving over land between water masses. This is congruent with the finding of a parautochthonous accumulation of bones from several individuals in an intertidal floodplain. *Sobrarbesiren* thus represents an intermediate stage in the evolution towards a fully aquatic life, between the amphibious quadrupedal prorastomids and the more aquatic quadrupedal protosirenids^4^. This contradicts our phylogenetic results, where Protosirenidae is constantly recovered as a sister taxon of *Sobrarbesiren* + Dugongidae. Further fossils and phylogenetic analyses are needed to test whether this represents a case of convergent evolution, where protosirenids and dugongids reduced their hind limbs independently during their adaptation to a fully aquatic lifestyle.

### Conclusions

We describe a new stem sirenian species, *Sobrarbesiren cardieli*, from the early middle Eocene (Lutetian, SBZ15) of the southern Pyrenees. This is the first adequately known quadrupedal sirenian from the northeastern Atlantic coast (Bay of Biscay, Spain) and the oldest sirenian from western Europe. It is represented by three skulls, cervical, dorsal, lumbar, sacral and caudal vertebrae, complete anterior, middle and posterior ribs, four scapulae, two hemipelvises, and fore and hind limb bones. This large collection of skeletal elements in different ontogenetic stages constitutes the most complete sample of an early middle Eocene sirenian in the Old World. Our phylogenetic analyses place *Sobrarbesiren cardieli* most parsimoniously as the sister taxon of a paraphyletic Dugongidae, which gives rise to Trichechidae. *Sobrarbesiren* is the only Eurasian sirenian that preserves terrestrially-functional pelvic girdle and hind limb bones. These indicate an intermediate state of adaptation to the aquatic lifestyle between other groups of quadrupedal sirenians, (namely the plesiomorphic pelvis and hind limbs of prorastomids, versus the slightly more derived limbs of the protosirenids). A further study of the functional morphology of sirenian hind limbs may be one of the keys to understanding the first stages of adaptation to aquatic life by these marine mammals.

## Methods

### Specimens

Fifty-six sirenian fossil bones were studied, including cranial and postcranial elements. The fossils were recovered in several field campaigns, using the usual methodology for macrovertebrate excavations. A metre-square grid system was applied and the spatial location of each element >1 cm in maximum dimension was recorded on a graph. The fossils are housed in the Museo de Ciencias Naturales de la Universidad de Zaragoza (Aragón, Spain).

The total body length of *Sobrarbesiren* was estimated using Sarko *et al*.^32^’s equation 9, which uses skull condylobasal length as a proxy.

### Phylogenetic analysis

To assess the phylogenetic position of *Sobrarbesiren cardieli* we included it in the most recently updated dataset for the group^1^. We also added seven Eocene sirenian taxa to this dataset, *Eotheroides lambondrano*, *Eotheroides sandersi*, *Eotheroides clavigerum*, *Prototherium veronense*, *Prototherium intermedium*, *Prototherium ausetanum* and *Libysiren sickenbergi*. *Eotheroides lambondrano* was coded based on Samonds *et al*.^33^ and the examination of a high-quality cast housed in the USNM. *Eotheroides sandersi* and *Eotheroides clavigerum* were coded based on the descriptions by Zalmout and Gingerich^25^, and pictures of the specimens provided by Iyad S. Zalmout. *Libysiren* was coded based on descriptions in Domning *et al*.^9^ and direct observations of the holotype. *Prototherium ausetanum*, *Prototherium veronense* and *Prototherium intermedium* were coded based on published descriptions and codifications of Balaguer y Alba^17^, Domning^23^ and Sickenberg^29^.

The resulting dataset includes a total of 50 taxa, including 48 sirenians with the proboscidean *Phosphatherium escuilliei* Gheerbrant *et al*.^34^, plus the multitaxic Elephantidae as the outgroup (Supplementary Information Data matrix 1). Following Springer *et al*.^1^, of the total of 74 parsimony-informative characters scored, characters 1, 6, 7, 16, 31, 36, 38, 44, 49, 50, 51, 60, 65, 67, 68, 70 and 71 were treated as additive (ordered) characters, whereas relative steps between states of characters 10, 27, and 47 are defined by step matrices. The name *Halitherium schinzii* was replaced by *Kaupitherium gruelli*, following Voss *et al*.^35^.

The dataset was analysed using the current version of TNT 1.5^36^. A heuristic search with 1000 replicates using Wagner trees as starting seeds, followed by branch swapping by tree-bisection-reconnection (TBR), holding ten trees per replicate, was conducted for each dataset. An additional round of TBR was performed, using the existing trees as starting trees, but this failed to recover any further trees. TNT does not support multiple outgroups but allows the re-rooting of trees after the search to a multiple-taxon outgroup by using the taxonomy option. This procedure was applied in order to resemble more closely the topology of the consensus shown by Springer *et al*.^1^. As advised by the documentation included with TNT, when using step matrices there is a possibility that re-rooting the trees to a taxonomic outgroup will influence the tree scores, but this was not the case for this dataset. Branch support was calculated using decay indexes with the script included in TNT, and 1000 replicates of bootstrap analysis. The resulting consensus recovers *Sobrabresiren cardieli* as sister taxon to a paraphyletic Dugongidae, which includes the family Trichechidae nested as the sister taxon of the clade including *Kaupitherium*, *Priscosiren*, *Caribosiren*, *Metaxytherium*, Hydrodamalinae and Dugonginae. To increase the resolution of the tree, *Eotheroides lambondrano*, known only by a partial skull and recovered as a wild card taxon, was a posteriori pruned from the consensus. The possible placements of *E*. *lambondrano* are shown by the stars in Fig. 11. The supplementary information attached to his manuscript includes a modified version of the matrix (Supplementary Information Data matrix 2). In this dataset, dummy parsimony-uninformative characters where added to the original dataset to alter the numbering sequence of the characters, as proposed by Domning^23^. This somewhat unorthodox numbering sequence has been kept in subsequent works for the purpose of consistency^1,9,24^. This modified matrix does produce the same exact results that the first dataset but simplifies the interpretation of the results on the light of previous work.

### Nomenclatural Acts

The electronic edition of this article conforms to the requirements of the amended International Code of Zoological Nomenclature, and hence the new names contained herein are available under that Code from the electronic edition of this article. This published work and the nomenclatural acts it contains have been registered in ZooBank, the online registration system for the ICZN. The ZooBank LSIDs (Life Science Identifiers) can be resolved and the associated information viewed through any standard web browser by appending the LSID to the prefix “http://zoobank.org/”. The LSID for this publication is: urn:lsid:zoobank.org:pub:17F24394-9E02-4794-99E9-DB85817EE54B.

### Data Availability

All data generated or analysed during this study are included in this published article and its supplementary information files. The TNT files are also available at MORPHOBANK http://morphobank.org/permalink/?P2628.

## Electronic supplementary material

Supplementary Information
