# Supplementary material for: First adequately-known quadrupedal sirenian from Eurasia (Eocene, Bay of Biscay, Huesca, northeastern Spain)
Source: Sci Rep. 2018 Mar 23;8:5127. doi: 10.1038/s41598-018-23355-w (PMC5865116; doi:10.1038/s41598-018-23355-w)
Supplement: Supplementary file 1 — Supplementary Information [file 41598_2018_23355_MOESM1_ESM.pdf]

SUPPLEMENTARY INFORMATION FOR

First adequately-known quadrupedal sirenian from Eurasia (Eocene, Bay of Biscay, Huesca, northeastern Spain)

*Ester Díaz-Berenguer[1], Ainara Badiola[2], Miguel Moreno-Azanza[3] & José Ignacio Canudo[1]

[1] Grupo Aragosaurus-IUCA, Facultad de Ciencias, Universidad de Zaragoza, 50009, Zaragoza, Spain.

[2] Dpto. Estratigrafía y Paleontología, Universidad del País Vasco (UPV/EHU), 48080, Bilbao, Spain.

3 Geobiotec, Dpto. de Ciências da Terra, Universidade Nova de Lisboa, 2829-526, Caparica, Portugal.

Correspondence and requests for materials should be addressed to E.D.B (ester.berenguer@gmail.com).

This pdf includes:

Geology and age

Supplementary figures

Supplementary measurements tables

Data matrix

References

Geology and age

The Castejón de Sobrarbe-41 sirenian fossil site (CS-41) is located near the small village of Castejón de Sobrarbe (Comarca de Sobrarbe, Huesca, northeastern Spain). Geologically, this sirenian bone bed crops out in the Ainsa Basin, which constitutes the westernmost part of the Cenozoic Jaca-Pamplona Basin in the South Pyrenean Central Unit[21]. During the Eocene, this area was a deep marine gulf (the Bay of Biscay) located between the Iberian Peninsula and Europe (Fig. 1a), which opened northwestwards into the Atlantic Ocean, delimited to the east by the emergence of reliefs associated with the Pyrenean orogen[22]. The Ainsa Basin originated during the early Eocene and is located between the anticlines of Boltaña and Mediano (Fig. 1b). The Eocene sediments range from Ypresian (Ilerdian) to Priabonian in age[21]. The Sobrarbe deltaic complex developeds in a syntectonic context confined by lateral thrust ramps and influenced by the formation of intrabasin anticlines[38]. Lithostratigraphically, the deltaic complex encompasses the upper part of the San Vicente Formation (lower slope marls and turbidite sandstones), the Sobrarbe Formation (delta-front sandstones and deltaic plain mudstones), and the lower to middle part of the Mondot Member of the Escanilla Formation (siliciclastic alluvial red beds)[38].

The CS-41 section shows the progradation of the Sobrarbe deltaic complex, from the delta-front facies, characterized by coarse-grained sandstones, to the deltaic and alluvial plain facies, dominated by fine-grained sandstones and mudstones, in which the CS-41 fossil site is located. CS-41 is a metre-thick level of mudstone, overlying laminated fine-grained sandstones with small burrowing traces. No lamination can be observed in the fossil-bearing bed, but the overlying siltstones present lamination and mud cracks, evidencing subaerial exposure.

The CS-41 fossiliferous level is located in the uppermost part of the Sobrarbe Formation and has been correlated with nearby sections dated by magnetostratigraphy and benthic foraminifera[21], placing CS-41 within the C19r chron and biozone SBZ15 (middle Lutetian, middle Eocene). The CS-41 fossil site is older than the sirenian fossil sites previously considered oldest in western Europe[20]. A total of 640 bone elements have been recovered from the 24 m^2 area excavated. To date, 300 disarticulated remains from at least six sirenian individuals, representing different ontogenetic stages, have been identified, together with turtle plates and bones, and scarce eusuchian crocodylomorph teeth. No other large vertebrates have been found. The microvertebrate fossil assemblage also includes chondrichthyan, squamate and micromammal teeth, abundant vertebrate eggshell fragments, invertebrates (gastropods and infaunal bivalves) and plant remains (wood fragments, and seeds). The fossil assemblage of CS-41 is a mixture of intertidal elements (razor-shell bivalves, Chondrichthyes) and continental elements (chelonian eggshells and squamates).

The sirenian fossil remains are complete and well preserved, but much fractured as a result of lithostatic compression and recent plant root bioturbation. The fossils are disarticulated, oriented but poorly sorted, with no signs of significant transport. This assemblage is interpreted as the infilling of an intertidal channel during a single energetic event, where all the remains dispersed over the tidal flat were trapped. Further analysis is needed to determine whether the death of the macrovertebrates was simultaneous due to a catastrophic event or whether their accumulation is related to attritional events.

Figure S1. Comparison of basicranial region of different Eocene sirenian skulls.

(a) *Sobrarbesiren cardieli* gen. et sp. nov. (MPZ 2017/1); (b) *Prorastomus sirenoides* (sketch based on holotype BMNH 44897, late early Eocene, Jamaica); (c) *Libysiren sickenbergi* (sketch based on Domning et al[9] figure 4, NHMUK M45675a, lower Lutetian, Libya); (d**)** *Eotheroides aegyptiacum* (sketch based on Abel[39] table (II) XXXI, figure 2, Lutetian, Egypt); (e) *"Halitherium" taulannense* (sketch based on holotype RGHP D040, Priabonian, France); (f**)** *Prototherium intermedium* (sketch based on Bizzotto[40] plate 4,b, skull 28998, Priabonian, Università di Padova, Italy). Anatomical abbreviations: bo, basioccipital**;** bs, basisphenoid; hf, hypoglossal foramina; oc, occipital condyle; so, supraoccipital**.** Institutional abbreviations: BMNH, British Museum (Natural History), London, UK; NHMUK, Fossil Vertebrate collection, Natural History Museum, London, UK; MPZ, Museo de Ciencias Naturales de la Universidad de Zaragoza, Zaragoza, Spain; RGHP**,** Réserve Géologique de Haute Provence, France.

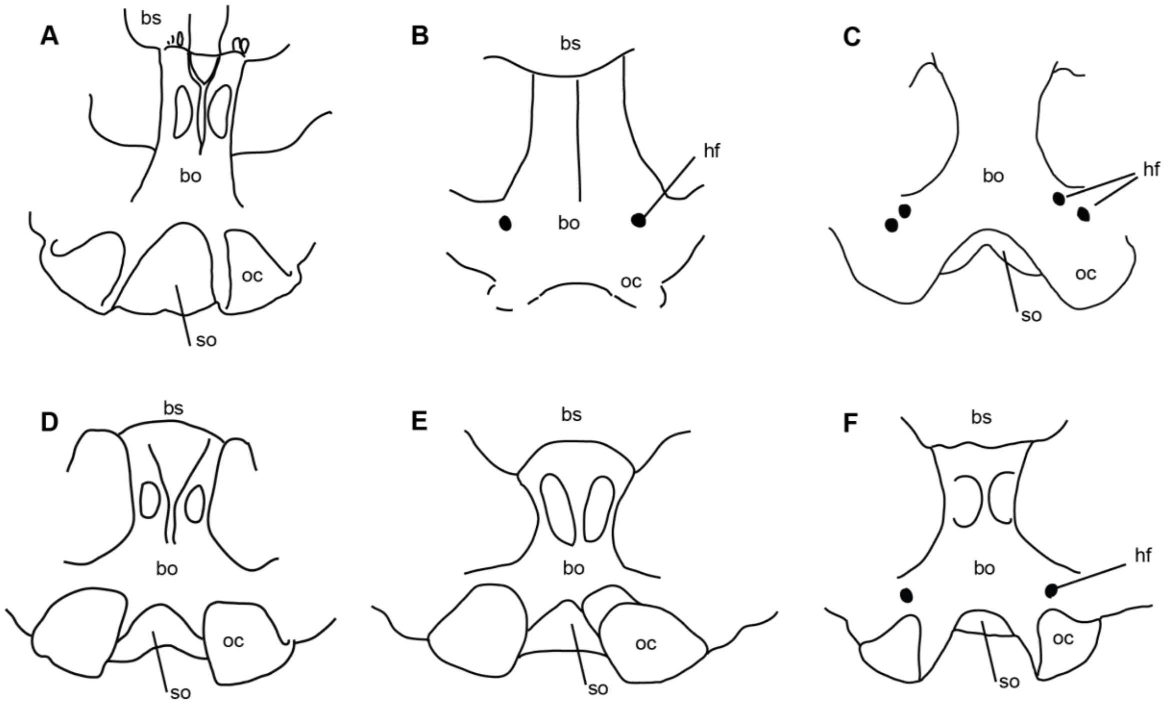

Figure S2. Skull of *Sobrarbesiren cardieli* gen. et sp. nov. (paratype, MPZ 2017/2).

Skull (MPZ 2017/2) photographs in lateral (a), dorsal (b), and ventral (c) views. Note: The M1-3 are taphonomically displaced from their original place.

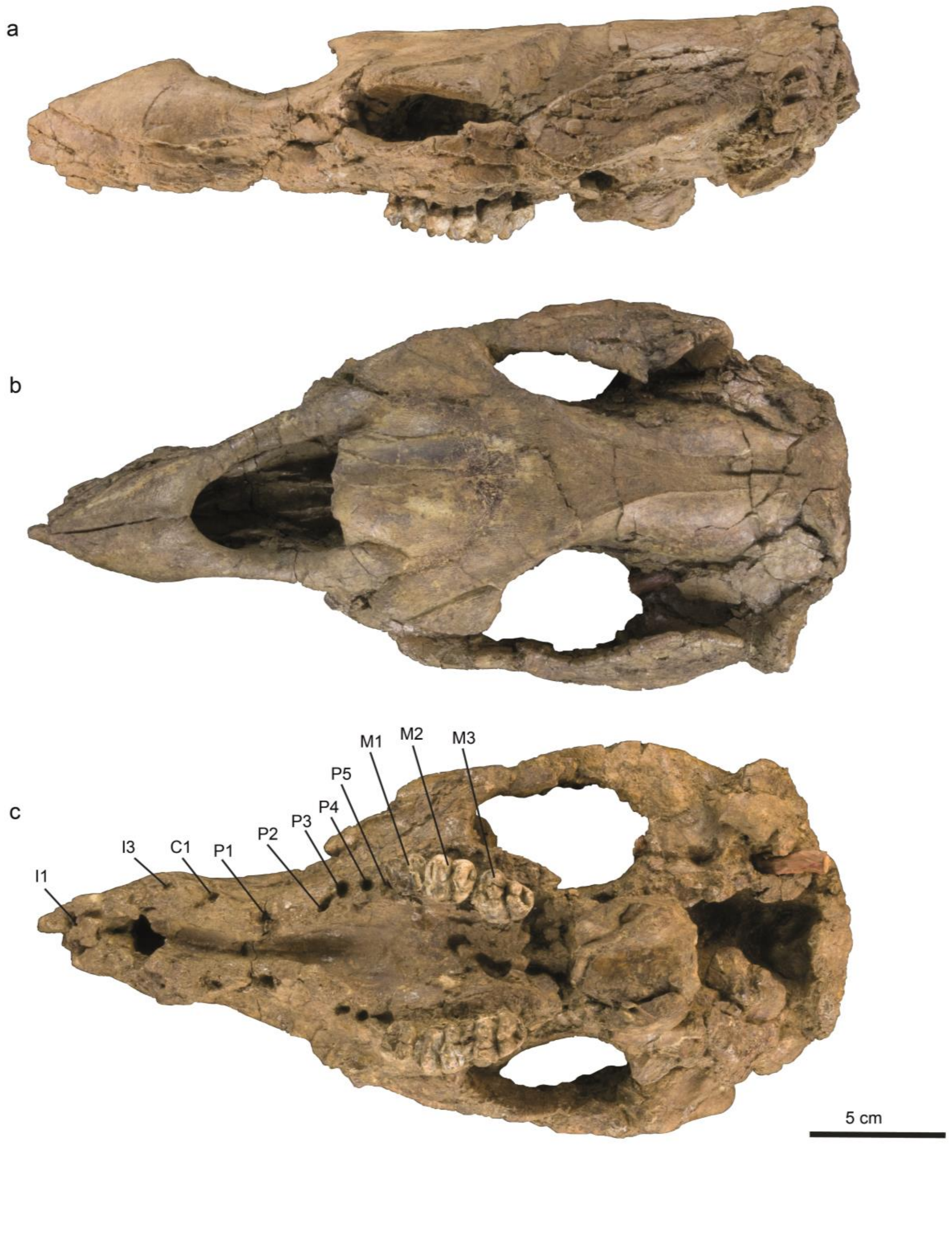

Figure S3. Vertebra and ribs of *Sobrarbesiren cardieli* gen. et sp. nov.

(a) First thoracic vertebra (MPZ 2017/10) in anterior view**.** (b) First right rib (MPZ 2017/22) in posterior view. (c) Anterior right rib (MPZ 2017/24) in anterior view.

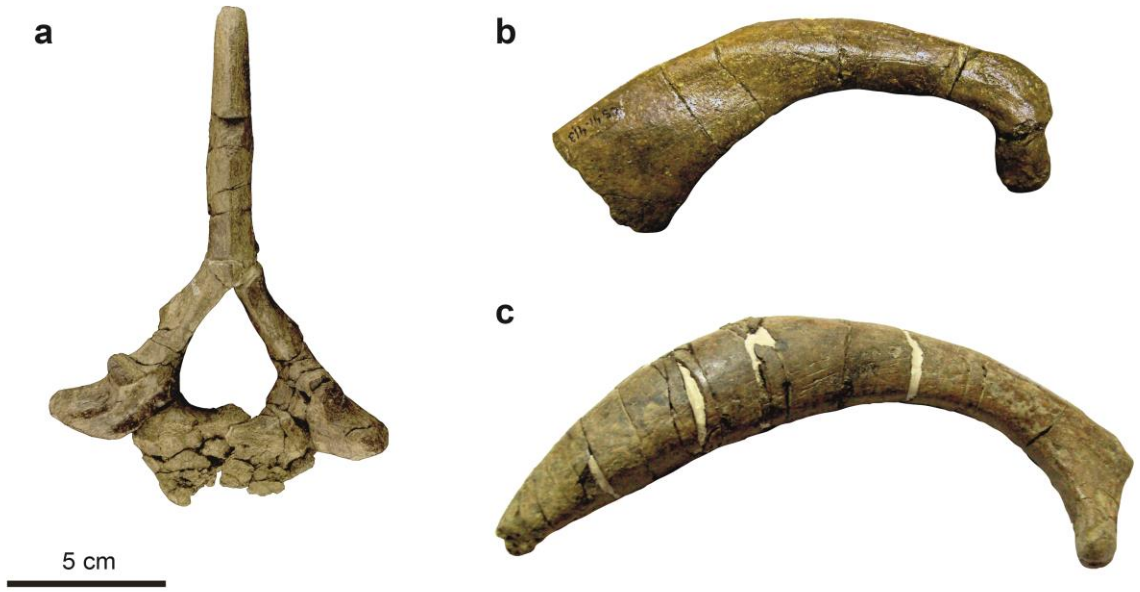

Table S1. **Measurements of skulls of *Sobrarbesiren cardieli* gen. et sp. nov., following Domning[41].**

Dimensions		Holotype skull MPZ 2017/1	Paratype skull MPZ 2017/2
**AB**	Condylobasal length	338	303
**ab**	Height of jugal below orbit	42e	x
**AH**	Length of premaxillary symphysis	105	64
**BI**	Rear of occipital condyles to anterior end of interfrontal suture	169	x
**CC´**	Zygomatic breadth	121*	150
**cc´**	Breadth across exoccipitals	87	x
**de**	Top of supraoccipital to ventral side of occipital condyles	103	x
**F**	Length of frontals—level of tips of supraorbital processes to frontoparietal suture	117	109
**FF´**	Breadth across supraorbital processes	120	115
**ff´**	Breadth across occipital condyles	68	x
**GG´**	Breadth of cranium at frontoparietal suture	65	60
**gg´**	Width of foramen magnum	28	x
**HI**	Length of mesorostral fossa	67	56
**hi**	Height of foramen magnum	43	x
**JJ´**	Width of mesorostral fossa	24*	42
**KL**	Maximum height of rostrum	71	52
**LFr**	Length of interfrontal suture	58	57
**MM´**	Posterior breadth of rostral masticating surface	31*	48
**no**	Anteroposterior length of zygomatic-orbital bridge	62	52
**OP**	Length of zygomatic process of squamosal	110	88
**OT**	Anterior tip of zygomatic process to rear edge of squamosal below mastoid foramen	126	x
**P**	Length of parietals—frontoparietal suture to rear of external occipital protuberance	97	68
**Pq**	Length of row of tooth alveoli DP5 to M3	69	x
**QR**	Anteroposterior length of root of zygomatic process of squamosal	46	44
**rr´**	Maximum width between labial edges of left and right alveoli	60*	83
**ST**	Length of cranial portion of squamosal	82	64
**ss´**	Breadth across sigmoid ridges of squamosal	122	x
**T**	Dorsoventral thickness of zygomatic orbital bridge	13	x
**tt´**	Anterior breadth of rostral masticating surface	19*	33
**UV**	Height of posterior part of cranial portion of squamosal	95	x
**WX**	Dorsoventral breadth of zygomatic processs	35	40

**yy´**	Maximum width between pterygoid processes	35	29
**YZ**	Length of jugal	139e	x
**Hso**	Height of supraoccipital	55	x
**Wso**	Width of supraoccipital	98	x
**RD**	Rostral deflection (degrees)	32°	x

Measurements are in millimetres. Maximum deviation of the digital calliper equals 0.02mm but measurements were rounded to the nearest 1 mm. (e) Estimated measurements. (x) Not measurable. (*) Distorted measurements.

Table S2. **Dentition measurements of *Sobrarbesiren cardieli* gen. et sp. nov. following Zalmout & Gingerich[25].**

**SPECIMEN**		**Crown length**	**Anterior width**	**Posterior width**
**Holotype skull MPZ 2017/1**	**P5 (Left)**	10	10,8	x
	**M1 (Left)**	18,2	16,7	14,9
	**M2 (Left)**	21,2	19	17,2
	**M3 (Left)**	25,4	20	15,6
	**M1 (Right)**	17,5	16,8	15,5
	**M2 (Right)**	21,3	20,7	17,7
	**M3 (Right)**	25	22,3	17,6
**Paratype skull MPZ 2017/2**	**M1 (Left)**	16,6	15,7	13,6
	**M2 (Left)**	19,6	19	16,4
	**M3 (Left)**	22,8	20,8	14,7
	**M1 (Right)**	15,7	16	14,3
	**M2 (Right)**	19,6	18,3	17,8
	**M3 (Right)**	23,4	17,5	14,2
**MPZ 2017/4**	**?I1**	15	x	x
**MPZ 2017/5**	**?I3**	6,5	x	x

Measurements are in millimetres. Maximum deviation of the digital calliper equals 0.02 mm, but measurements were rounded to the nearest 0,1 mm. (x) Not measurable.

Table S3. **Selected measurements of vertebrae of *Sobrarbesiren cardieli* gen. et sp. nov**. **following Zalmout & Gingerich[25].**

SPECIMEN	Total height	Total width	Centrum length	Centrum width	Centrum height
Atlas (MPZ 2017/6)	> 62	126	x	x	x
Axis (MPZ 2017/7)	90	38	51	21	30
Cervical vertebral body (MPZ 2017/8)	x	x	15	31	28
Anterior thoracic vertebra (MPZ 2017/10)	155	108	18	53	27
Thoracic vertebra (MPZ 2017/12)	169	122	43	66	35
Anterior lumbar vertebra (MPZ 2017/16)	130	137	40	66	44
Sacral vertebra (MPZ 2017/18)	x	198	45	70	38
Anterior caudal vertebra (MPZ 2017/20)	113	173	53	70	40

Measurements are in millimetres. Maximum deviation of the digital calliper equals 0.02mm but measurements were rounded to the nearest 1 mm. (x) Not measurable.

Table S4. **Selected measurements of ribs of *Sobrarbesiren cardieli* gen. et sp. nov**. **following Zalmout & Gingerich[25].**

SPECIMEN	TL	MAWM	MLWM
Left first rib (MPZ 2017/22)	103	22	25
Right anterior rib (MPZ 2017/23)	189	41	42
Rigth anterior rib (MPZ 2017/24)	171	35	40
Rigth anterior rib (MPZ 2017/25)	189	36	40
Left anterior rib (MPZ 2017/26)	215	44	45
Right medial rib (MPZ 2017/27)	300	40	27
Rigth medial rib (MPZ 2017/28)	363	35	31
Rigth medial rib (MPZ 2017/29)	310	27	17
Right medial rib (MPZ 2017/30)	340	33	22
Right medial rib (MPZ 2017/31)	297	36	24
Right medial rib (MPZ 2017/32)	264	36	26
Right posterior rib (MPZ 2017/33)	275	23	21
Left anterior rib (MPZ 2017/34)	168	37	45
Left medial rib (MPZ 2017/35)	260	34	29
Left medial rib diaphysis (MPZ 2017/36)	208	28	18
Left posterior rib (MPZ 2017/37)	255	20	13
Left posterior rib (MPZ 2017/38)	287	22	14
Left posterior rib (MPZ 2017/39)	295	25	17

Measurements are in millimetres. Maximum deviation of the digital calliper equals 0.02 mm, but measurements were rounded to the nearest 1 mm. Abbreviations: MAWM, maximum anteroposterior width of midshaft; MLWM, maximum mediolateral width of midshaft; TL, total length (straight line).

Table S5. **Selected measurements of scapulae of *Sobrarbesiren cardieli* gen. et sp. nov**. **following Zalmout & Gingerich[25].**

SPECIMEN	Total length	Total width	Infraspinous fossa breath	Glenoid cavity breadth
Left scapula (MPZ 2017/40)	253	90	69	45
Left scapula (MPZ 2017/41)	244	109*	83*	45
Left scapula (MPZ 2017/42)	245	95	62	43
Left scapula (MPZ 2017/43)	234	93	65	x

Measurements are in millimetres. Maximum deviation of the digital calliper equals 0.02 mm, but measurements were rounded to the nearest 1 mm. (x) Not measurable. (*) Distorted measurements.

Table S6. **Selected measurements of forelimb bones of *Sobrarbesiren cardieli* gen. et sp. nov**. **following Zalmout & Gingerich[25].**

SPECIMENS	Left humerus (MPZ 2017/27)	Right ulna (MPZ 2017/46)
**Total lenght**	206	147
**Maximum breadth of the proximal end**	67	20
**Maximum breadth of the distal end**	64	30
**Head length**	46	x
**Head width**	50	x
**Width of the biccipital groove**	30	x
**Trochlea breadth**	40	x

**Trochlea height**	32	x
**Depth of the olecranial fossa**	14	x

Measurements are in millimetres. Maximum deviation of the digital calliper equals 0.02 mm, but measurements were rounded to the nearest 1 mm. (x) Not measurable.

Table S7. Selected measurements of innominate bones of *Sobrarbesiren cardieli* gen. et sp. nov. following Zalmout & Gingerich[25].

SPECIMEN	Total length	Pubic symphysis length	Acetabulum diameter	Acetabulum depth
Left innominate bone (MPZ 2017/47)	240	35	32	21

Measurements are in millimetres. Maximum deviation of the digital calliper equals 0.02 mm, but measurements were rounded to the nearest 1 mm.

Table S8. Selected measurements of hindlimb bones of *Sobrarbesiren cardieli* gen. et sp. nov. following Zalmout & Gingerich[25].

SPECIMEN	Total length	Maximum breadth of proximal end	Maximum breadth of distal end
Patella (MPZ 2017/51)	28	x	x
Left femur (MPZ 2017/53)	134	42	33
Left fibula (MPZ 2017/56)	65	11	9

Measurements are in millimetres. Maximum deviation of the digital calliper equals 0.02 mm, but measurements were rounded to the nearest 1 mm. (x) Not measurable.

DATA MATRIX 1

nstates 8 ;

taxna + 100

taxname[!

TAXONOMY=;

xread

'Data from Diaz-Berenguer et al. 2018.

Based on an updated version of Springer et al. 2015.'

74 50

&[num]

Anomotherium_langewieschei____@sirenia

???????????0001000100102210?2??????????1111113112?100?????1211011??2??????

Ashokia_antiqua____@sirenia

?01?100?001000?00?10000120001000?0?1???0?11?1???????????????0?????????????

Bharatisiren_indica____@sirenia

20110100?0013100001000?02110102010?1?20121111?3?11111?020?122101????????

??

Bharatisiren_kachchhensis____@sirenia

21110100100131021011000021101020101112011111?????????022?122101?????????

?

Caribosiren_turneri____@sirenia

20110100?0011000001????0211010?0?0?1110111111??????????0??122101??????????

Corystosiren_varguezi____@sirenia

2?11????????32?2011?01?02010?02?10?11?????1?1????????1023112?101??????????

Crenatosiren_olseni____@sirenia

2011010000013100001001002110101000111?01111114312111100100122101?012?0?

101

Dioplotherium_allisoni____@sirenia

22110101100131?21110020?201010201111101211114302111110221122101?????0?

???

Dioplotherium_manigaulti____@sirenia

2211010010013102011102 0??1????20111112012 1111????????1022?122101?????????

?

Domningia_sodhae____@sirenia

22110100000132121010010021101021101212011112 1431?1?1?10221122101????????

??

Dugong_dugon____@sirenia

2011021100013202111111002130102110111111111114312111110201122101?01210

2111

Dusisiren_jordani____@sirenia

2011110001011000001114002120102100020101111113212 1101?10??122101?012?02

111

ECOCHM_2491____@sirenia

22110?00000102021010110021101120111 1??0121111????????1022112?101?????????

?

Elephantidae____@proboscidea

00001210000000?000100?100000?000000100100000?0000010?1???02210020?0??00
10

Eotheroides_aegyptiacum____@sirenia

?011000000?000000100100211?00?0???000?111111?11???0??????0101011???????
?

Kaupitherium_gruelli____@sirenia

2011010000000000001001002110101000?011011111141021101001001211011012?02
111

Halitherium_taulannense____@sirenia

2011010000000000001001002110100000?011011111111021100001000101011???102
1??

Hydrodamalis_cuestae____@sirenia

20111110020110000011140031201021000211111111132121101?10??122101?0?2?02
1??

Hydrodamalis_gigas____@sirenia

20111110020120000011150031001021000201111111132120201?10??122101?012102
111

Kutchisiren_cylindrica____@sirenia

211101000001310100110???211?11201?1102012111?1302111110221122101????????
??

Metaxytherium_arctodites____@sirenia

2011110000011000001112002110102100010101111114312111100000122101?01210

2111

Metaxytherium_crataegense____@sirenia

2011110000011000001112002110102000011101111114312111100000122101?012?02

111

Metaxytherium_floridanum____@sirenia

2011111000011000001112002110102000011101111114312111100000122101?01210

2111

Metaxytherium_krahuletzi____@sirenia

20111100000?1000001112002110101?000?1??11111143121111000??122101?012?02

111

Metaxytherium_medium____@sirenia

2011110000011000001112002110102000011101111114312111100000122101?012?02

1??

Metaxytherium_serresii____@sirenia

2011110000011000001112002110102000011111111114312111100100122101?012?02

111

Miosiren_kocki____@sirenia

101101010?1010100011010221002020000101011111 1???????0020012110110?2??2?

??

Nanosiren_garciae____@sirenia

2?110?1000?1?101?11111102130101?001???0121121?312?11??00??122101?????0??0

1

Nanosiren_sanchezi____@sirenia

20110?100001?101?11111102130101?001???0???121????????00000122101??????????

Pezosiren_portelli____@sirenia

10111?0????000?000000000000010????????00?0011110100000010001000100010000

11

Phosphatherium_escuillei____@proboscidea

0000100?0?1000?0000?000??00?0?00000?000000000?0000?00000000001000????????

?

Prorastomus_sirenoides____@sirenia

101110000?00000000?000001000000000?000000000110000000?0?0?0000010???????

??

Protosiren_fraasi____@sirenia

101100000?1000000010000110010000000??000001111210??10000000000100011???????

??

Protosiren_smithae____@sirenia

10110000?01100000010110110010000000??0000011?121011101?020?0100011002?00

000

Rytiodus_capgrandi____@sirenia

221???0????131?2001?01?02?10?020?0??1???1?111????????10231???101??????????

Rytiodus_heali____@sirenia

2211020000013102001011OO?010?020101112011111?31??11?10231122101????????

??

Trichechus_inunguis____@sirenia

10111101001100000010031121012000OO?30111111213202210O?10??122111?123012

112

Trichechus_manatus____@sirenia

101111010011000100100111210121OOOO?301111112132022101?10??122111?123022

112

Trichechus_senegalensis____@sirenia

101111010011000100100101210121OOOO?301111112132022101?10??122111?123022

112

Metaxytherium_albifontanum____@sirenia

2011010000011000001112002110101000011101111?14312?11100000122101?012102

111

Priscosiren_atlantica____@sirenia

2011010000010000001000002110102000011101111114312110 1?000?122101??12???

???

Xenosiren_yucateca____@sirenia

?21?0?2110?1310201????????10?01?111???0??????????????102?1???101??????????

Sobrarbesiren_cardieli____@sirenia

20111000000000?0001?01?0211000?000?00?00111?????????00010010011??1?0000

1

Eotheroides_lambondrano____@sirenia

2111?11?000000?00010??????10?010?1011?0???1???????????0??????????????????

Eotheroides_sandersi____@sirenia

1[01]11?11???00?0?0001001001110?000?1011001??12?110????0?0???010101100 2?0

1?1[01]

Eotheroides_clavigerum____@sirenia

1[01]1??11???0000?000100?0??110?000??01??01??1??11021?000000?01010110?2?02

?1[01]

Prototherium_veronense____@sirenia

10111100?100000000110[01]???1101000001?01?1[12]11???20101000001001010 11??

??0??0?

Prototherium_intermedium____@sirenia

20110?00?100000000100[01]???110?000000???011????1021100001??010?011????0?

?0?

Prototherium_ausetanum____@sirenia

10110??0?00000?0001101?0?110?00?0000?001111???????????????101001?????????

Libysiren_sickenbergi____@sirenia

101101000?1000?00010010120110000 00?12??00?1?1210?1100?00??0100011????????

?

;

Ccode

```
  +[/1  0       -[/1  1       -[/1  2       -[/1  3       -[/1  4
  +[/1  5       +[/1  6       -[/1  7       -[/1  8       ([/1  9
  -[/1  10      -[/1  11      -[/1  12      -[/1  13      -[/1  14
  +[/1  15      -[/1  16      -[/1  17      -[/1  18      -[/1  19
  -[/1  20      -[/1  21      -[/1  22      -[/1  23      -[/1  24
  -[/1  25      ([/1  26      -[/1  27      -[/1  28      -[/1  29
  +[/1  30      -[/1  31      -[/1  32      -[/1  33      -[/1  34
  +[/1  35      -[/1  36      +[/1  37      -[/1  38      -[/1  39
  -[/1  40      -[/1  41      -[/1  42      +[/1  43      -[/1  44
  -[/1  45      ([/1  46      -[/1  47      +[/1  48      +[/1  49
  +[/1  50      -[/1  51      -[/1  52      -[/1  53      -[/1  54
  -[/1  55      -[/1  56      -[/1  57      -[/1  58      +[/1  59
  +[/1  60      -[/1  61      -[/1  62      -[/1  63      +[/1  64
  -[/1  65      +[/1  66      +[/1  67      -[/1  68      +[/1  69
  +[/1  70      -[/1  71      -[/1  72      -[/1  73    ;
cost 9 = 0>1 1 0>2 1 1>0 1 1>2 2 2>0 1 2>1 2;
cost 26 = 0>1 1 0>2 2 0>3 2 1>0 1 1>2 1 1>3 1 2>0 2 2>1 1 2>3 2 3>0 2 3>1 1 3>2 2;
cost 46 = 0>1 2 0>2 1 0>3 2 1>0 2 1>2 1 1>3 2 2>0 1 2>1 1 2>3 1 3>0 2 3>1 2 3>2 1;
cnames
{0 Rostrum;
```

{1 Nasal_processes_of_premaxillae;

{2 External_nares;

{3 Premaxillary_symphysis;

{4 Level_of_zygomatic-orbital_bridge_of_maxilla;

{5 'Infraorbital_foramen';

{6 Length_of_zygomatic-orbital_bridge_of_Maxilla;

{7 Palate;

{8 Infraorbital_canal;

{9 Edges_of_zygomatic-orbital_bridge_of_maxilla;

{10 Anterior_palate;

{11 Nasals;

{12 Supraorbital_process_of_frontal;

{13 Nasal_incisure_at_posterior_end_of_mesorostral_fossa;

{14 Frontal,_lamina_orbitalis;

{15 Frontal_roof_shape;

{16 Supraorbital_process_of_frontal;

{17 Frontal_roof_bosses;

{18 Sagittal_crest;

{19 Supraoccipital;

{20 Union_of_exoccipitals;

{21 Exoccipital,_dorsolateral_border;

{22 'Hypoglossal_foramen';

{23 'Postympanic_process_of_squamosal';

{24 Squamosal,_sigmoid_ridge;

{25 Cranial_portion_of_squamosal;

{26 Processus_retroversus_of_squamosal;

{27 'Outline_of_zygomatic_process_of_squamosal';

{28 'External_auditory_meatus_of_squamosal';

{29 'Zygomatic_process_of_squamosal';

{30 'Ventral_extremity_of_jugal';

{31 'Preorbital_process_of_jugal contact with;

{32 'Preorbital_process_of_jugal';

{33 Zygomatic_process_of_jugal;

{34 'Ventral_rim_of_orbit';

{35 Lacrimal_foramen;

{36 Lacrimal-premaxilla_contact;

{37 Posterior_border_of_palatine;

{38 Anterior_border_of_palatine;

{39 'Alisphenoid_canal';

{40 'Pterygoid_fossa';

{41 'Foramen_ovale';

{42 Sphenopalatine_region;

{43 Periotic;

{44 Mastoid_portion_of_periotic;

{45 Mandibular_symphysis;

{46 Ventral_border_of_horizontal_mandibular_ramus;

{47 'Accesory_mental_foramina';

{48 'Posterior_border_of_mandible';

{49 Anterior_border_of_coronoid_process;

{50 Mandibular_dental_capsule;

{51 Horizontal_ramus_of_mandible;

{52 Ventral_border_of_horizontal_ramus_of_mandible;

{53 First_upper_incisor;

{54 First_upper_incisor;

{55 Depth_of_I1_alveolus;

{56 Cross_section_of_I1_crown;

{57 First_upper_incisor;

{58 Second_and_third_upper_incisors,_first_through_third_lower_incisors;

{59 Canines;

{60 Premolars_1-4;

{61 Permanent_premolar_5;

{62 Supernumerary_molars;

{63 Postcanine_dental_formulaCharacter_64;

{64 Permanent_premolars;

{65 Cervical_vertebrae;

{66 Lumbar_vertebrae;

{67 Sacrum;

{68 'Posterior_caudal_vertebrae';

{69 Bicipital_groove_of_humerus;

{70 Pubis;

{71 Tibia;

{72 Humerus;

{73 Deltoid_process_of_humerus;

;

;

Ancstates

-0 -1 -2 -3 -4 -5 -6 -7 -8 -9

-10 -11 -12 -13 -14 -15 -16 -17 -18 -19

-20 -21 -22 -23 -24 -25 -26 -27 -28 -29

-30 -31 -32 -33 -34 -35 -36 -37 -38 -39

-40 -41 -42 -43 -44 -45 -46 -47 -48 -49

-50 -51 -52 -53 -54 -55 -56 -57 -58 -59

-60 -61 -62 -63 -64 -65 -66 -67 -68 -69

-70 -71 -72 -73 ;

smatrix =0 (StepMatrix9)

0/1 1 0/2 1 0/3 1 0/4 1 0/5 1 1/2 2

1/3 1 1/4 1 1/5 1 2/3 1 2/4 1 2/5 1

3/4 1 3/5 1 4/5 1 ;

smatrix =1 (StepMatrix26)

0/1 1 0/2 2 0/3 2 0/4 1 0/5 1 1/2 1

1/3 1 1/4 1 1/5 1 2/3 2 2/4 1 2/5 1

3/4 1 3/5 1 4/5 1 ;

smatrix =2 (StepMatrix46)

0/1 2 0/2 1 0/3 2 0/4 1 0/5 1 1/2 1

1/3 2 1/4 1 1/5 1 2/3 1 2/4 1 2/5 1

3/4 1 3/5 1 4/5 1 ;

xgroup

;

agroup

;

taxcode

+0 +1 +2 +3 +4 +5 +6 +7

+8 +9 +10 +11 +12 +13 +14 +15

+16 +17 +18 +19 +20 +21 +22 +23

+24 +25 +26 +27 +28 +29 +30 +31

+32 +33 +34 +35 +36 +37 +38 +39

+40 +41 +42 +43 +44 +45 +46 +47

+48 +49

;

blocks 0;

Outgroup 13;

Outgroup[proboscidea;

Hold 50000;

proc/;

Data Matrix 2

taxonomy=;

nstates 32 ;

taxna + 100;

taxname[!;

xread

'Data from Diaz-Berenguer et al. 2018. Based on an updated version of Springer et al. 2015.

Additional dummy characters added as non-parsimony informative characters

(scored as A)to adapt character numbers to Domning 1994'

223 50

&[num]

Anomotherium_langewieschei____@sirenia

AAA?AA?A?A??A???AAAA?A??AAAAAAA0AAAA001AAA00A0AAAAA1AAA

AAAAAAAAA0A0AAA1A022A10AAA?2A???A????A?AAA?A?A111A1AAAAAA

AAA1AA1AA311A2?100AAAAAAA?A????1211AAA0AAAA1A1AAAAAAAAAA

AAAAAAAAAAAAAAAAAAAAAAAAAAAAAAAAA?AAA?2A?AAAAA?A?AAA

A???

Ashokia_antiqua____@sirenia

AAA?AA0A1A?1A00?AAAA0A01AAAAAAA0AAAA00?AAA00A?AAAAA1AAA

AAAAAAAAA0A0AAA0A012A00AAA01A000A?0?1A?AAA?A?A0?1A1AAAAA

AAAA?AA1AA???A?????AAAAAAA?A??????0?AAA?AAAA?A?AAAAAAAAAA

AAAAAAAAAAAAAAAAAAAAAAAAAAAAAAAAA?AAA??A?AAAAA?A?AAA

A???

Bharatisiren_indica____@sirenia

AAA2AA0A1A10A100AAAA?A00AAAAAAA1AAAA310AAA00A0AAAAA1AA

AAAAAAAAAA0A0AAA0A?02A11AAA01A020A10?1A?AAA2A0A121A1AAAA

AAAAA1AA1AA?3?A11111AAAAAAA?A020?1221AAA0AAAA1A?AAAAAAAA

AAAAAAAAAAAAAAAAAAAAAAAAAAAAAAAAAA?AAA??A?AAAAA?A?A

AAA???

Bharatisiren_kachchhensis____@sirenia

AAA2AA1A1A10A100AAAA1A00AAAAAAA1AAAA310AAA21A0AAAAA1AA

AAAAAAAAAA1A0AAA0A002A11AAA01A020A1011A1AAA2A0A111A1AAAA

AAAAA1AA1AA???A?????AAAAAAA?A022?1221AAA0AAAA1A?AAAAAAAA

AAAAAAAAAAAAAAAAAAAAAAAAAAAAAAAAAA?AAA??A?AAAAA?A?A

AAA???

Caribosiren_turneri____@sirenia

AAA2AA0A1A10A100AAAA?A00AAAAAAA1AAAA100AAA00A0AAAAA1AA

AAAAAAAAAA?A?AAA?A?02A11AAA01A0?0A?0?1A1AAA1A0A111A1AAAA

AAAAA1AA1AA???A?????AAAAAAA?A?0??1221AAA0AAAA1A?AAAAAAAA

AAAAAAAAAAAAAAAAAAAAAAAAAAAAAAAAAA?AAA??A?AAAAA?A?A

AAA???

Corystosiren_varguezi____@sirenia

AAA2AA?A1A1?A???AAAA?A??AAAAAAA?AAAA32?AAA20A1AAAAA1AAA

AAAAAAAAA?A0AAA1A?02A01AAA0?A02?A10?1A1AAA?A?A???A1AAAAAA

AAA?AA1AA???A?????AAAAAAA1A023112?1AAA0AAAA1A?AAAAAAAAAA

AAAAAAAAAAAAAAAAAAAAAAAAAAAAAAAA?AAA??A?AAAAA?A?AAA

A???

Crenatosiren_olseni____@sirenia

AAA2AA0A1A10A100AAAA0A00AAAAAAA1AAAA310AAA00A0AAAAA1AA

AAAAAAAAAA0A0AAA1A002A11AAA01A010A0011A1AAA?A0A111A1AAAA

AAAAA1AA1AA431A21111AAAAAAA0A01001221AAA0AAAA1A?AAAAAAA

AAAAAAAAAAAAAAAAAAAAAAAAAAAAAAAAAAA0AAA12A?AAAAA0A?

AAAA101

Dioplotherium_allisoni____@sirenia

AAA2AA2A1A10A101AAAA1A00AAAAAAA1AAAA31?AAA21A1AAAAA1AA

AAAAAAAAAA0A0AAA2A0?2A01AAA01A020A1111A1AAA1A0A121A1AAAA

AAAAA1AA1AA430A21111AAAAAAA1A02211221AAA0AAAA1A?AAAAAAA

AAAAAAAAAAAAAAAAAAAAAAAAAAAAAAAAAAA?AAA??A?AAAAA0A?

AAAA???

Dioplotherium_manigaulti____@sirenia

AAA2AA2A1A10A100AAAA1A00AAAAAAA1AAAA310AAA20A1AAAAA1AA

AAAAAAAAAA1A0AAA2A0??A1?AAA??A?20A1111A1AAA2A0A121A1AAAA

AAAAA1AA1AA???A?????AAAAAAA1A022?1221AAA0AAAA1A?AAAAAAAA

AAAAAAAAAAAAAAAAAAAAAAAAAAAAAAAAAAAA?AAA??A?AAAAA?A?A

AAA???

Domningia_sodhae____@sirenia

AAA2AA2A1A10A100AAAA0A00AAAAAAA1AAAA321AAA21A0AAAAA1AA

AAAAAAAAAA0A0AAA1A002A11AAA01A021A1012A1AAA2A0A111A1AAAA

AAAAA2AA1AA431A?1?1?AAAAAAA1A02211221AAA0AAAA1A?AAAAAAAA

AAAAAAAAAAAAAAAAAAAAAAAAAAAAAAAAAAAA?AAA??A?AAAAA?A?A

AAA???

Dugong_dugon____@sirenia

AAA2AA0A1A10A211AAAA0A00AAAAAAA1AAAA320AAA21A1AAAAA1AA

AAAAAAAAAA1A1AAA1A002A13AAA01A021A1011A1AAA1A1A111A1AAAA

AAAAA1AA1AA431A21111AAAAAAA1A02011221AAA0AAAA1A?AAAAAAA

AAAAAAAAAAAAAAAAAAAAAAAAAAAAAAAAAAAAA0AAA12A1AAAAA0A

2AAAA111

Dusisiren_jordani____@sirenia

AAA2AA0A1A11A100AAAA0A10AAAAAAA1AAAA100AAA00A0AAAAA1AA

AAAAAAAAAA1A1AAA4A002A12AAA01A021A0002A0AAA1A0A111A1AAAA

AAAAA1AA1AA321A21101AAAAAAA?A10??1221AAA0AAAA1A?AAAAAAAA

AAAAAAAAAAAAAAAAAAAAAAAAAAAAAAAAAAAA0AAA12A?AAAAA0A2

AAAA111

ECOCHM_2491____@sirenia

AAA2AA2A1A10A?00AAAA0A00AAAAAAA1AAAA020AAA21A0AAAAA1AA

AAAAAAAAAA0A1AAA1A002A11AAA01A120A1111A?AAA?A0A121A1AAAA

AAAAA1AA1AA???A?????AAAAAAA1A022112?1AAA0AAAA1A?AAAAAAAA

AAAAAAAAAAAAAAAAAAAAAAAAAAAAAAAAAA?AAA??A?AAAAA?A?A

AAA???

Elephantidae____@proboscideaa

AAA0AA0A0A01A210AAAA0A00AAAAAAA0AAAA00?AAA00A0AAAAA1AA

AAAAAAAAAA0A0AAA?A100A00AAA0?A000A0001A0AAA0A1A000A0AAAA

AAAAA0AA0AA?00A00010AAAAAAA?A1???0221AAA0AAAA0A2AAAAAAAA

AAAAAAAAAAAAAAAAAAAAAAAAAAAAAAAAAA0AAA?0A?AAAAA?A0A

AAA010

Eotheroides_aegyptiacum____@sirenia

AAA?AA0A1A10A000AAAA0A0?AAAAAAA0AAAA000AAA00A0AAAAA1AAA

AAAAAAAAA0A0AAA1A002A11AAA?0A0?0A???0A0AAA0A?A111A1AAAAA

AAAA1AA1AA?11A???0?AAAAAAA?A????0101AAA0AAAA1A1AAAAAAAAA

AAAAAAAAAAAAAAAAAAAAAAAAAAAAAAAAA?AAA??A?AAAAA?A?AA

AA???

Kaupitherium_gruelli____@sirenia

AAA2AA0A1A10A100AAAA0A00AAAAAAA0AAAA000AAA00A0AAAAA1AA

AAAAAAAAAA0A0AAA1A002A11AAA01A010A00?0A1AAA1A0A111A1AAAA

AAAAA1AA1AA410A21101AAAAAAA0A01001211AAA0AAAA1A1AAAAAAA

AAAAAAAAAAAAAAAAAAAAAAAAAAAAAAAAAAA0AAA12A?AAAAA0A

2AAAA111

Halitherium_taulannense____@sirenia

AAA2AA0A1A10A100AAAA0A00AAAAAAA0AAAA000AAA00A0AAAAA1AA

AAAAAAAAAA0A0AAA1A002A11AAA01A000A00?0A1AAA1A0A111A1AAAA

AAAAA1AA1AA110A21100AAAAAAA0A01000101AAA0AAAA1A1AAAAAAA

AAAAAAAAAAAAAAAAAAAAAAAAAAAAAAAAAAA?AAA??A1AAAAA0A2
AAAA1??

Hydrodamalis_cuestae____@sirenia

AAA2AA0A1A11A110AAAA0A20AAAAAAA1AAAA100AAA00A0AAAAA1AA
AAAAAAAAAA1A1AAA4A003A12AAA01A021A0002A1AAA1A1A111A1AAAA
AAAAA1AA1AA321A21101AAAAAAA?A10??1221AAA0AAAA1A?AAAAAAAA
AAAAAAAAAAAAAAAAAAAAAAAAAAAAAAAAAA0AAA?2A?AAAAA0A2A
AAA1??

Hydrodamalis_gigas____@sirenia

AAA2AA0A1A11A110AAAA0A20AAAAAAA1AAAA200AAA00A0AAAAA1AA
AAAAAAAAAA1A1AAA5A003A10AAA01A021A0002A0AAA1A1A111A1AAAA
AAAAA1AA1AA321A20201AAAAAAA?A10??1221AAA0AAAA1A?AAAAAAAA
AAAAAAAAAAAAAAAAAAAAAAAAAAAAAAAAAA0AAA12A1AAAAA0A2
AAAA111

Kutchisiren_cylindrica____@sirenia

AAA2AA1A1A10A100AAAA0A00AAAAAAA1AAAA310AAA10A0AAAAA1AA
AAAAAAAAAA1A0AAA?A??2A11AAA?1A120A1?11A0AAA2A0A121A1AAAA
AAAAA1AA?AA130A21111AAAAAAA1A02211221AAA0AAAA1A?AAAAAAA
AAAAAAAAAAAAAAAAAAAAAAAAAAAAAAAAAAA?AAA??A?AAAAA?A?
AAAA???

Metaxytherium_arctodites____@sirenia

AAA2AA0A1A11A100AAAA0A00AAAAAAA1AAAA100AAA00A0AAAAA1AA
AAAAAAAAAA1A1AAA2A002A11AAA01A021A0001A0AAA1A0A111A1AAAA
AAAAA1AA1AA431A21111AAAAAAA0A00001221AAA0AAAA1A?AAAAAAA

AAAAAAAAAAAAAAAAAAAAAAAAAAAAAAAAAAA0AAA12A1AAAAA0A

2AAAA111

Metaxytherium_crataegense____@sirenia

AAA2AA0A1A11A100AAAA0A00AAAAAAA1AAAA100AAA00A0AAAAA1AA

AAAAAAAAAA1A1AAA2A002A11AAA01A020A0001A1AAA1A0A111A1AAAA

AAAAA1AA1AA431A21111AAAAAAA0A00001221AAA0AAAA1A?AAAAAAA

AAAAAAAAAAAAAAAAAAAAAAAAAAAAAAAAAAA0AAA12A?AAAAA0A

2AAAA111

Metaxytherium_floridanum____@sirenia

AAA2AA0A1A11A110AAAA0A00AAAAAAA1AAAA100AAA00A0AAAAA1AA

AAAAAAAAAA1A1AAA2A002A11AAA01A020A0001A1AAA1A0A111A1AAAA

AAAAA1AA1AA431A21111AAAAAAA0A00001221AAA0AAAA1A?AAAAAAA

AAAAAAAAAAAAAAAAAAAAAAAAAAAAAAAAAAA0AAA12A1AAAAA0A

2AAAA111

Metaxytherium_krahuletzi____@sirenia

AAA2AA0A1A11A100AAAA0A00AAAAAAA?AAAA100AAA00A0AAAAA1AA

AAAAAAAAAA1A1AAA2A002A11AAA01A01?A000?A1AAA?A?A111A1AAAA

AAAAA1AA1AA431A21111AAAAAAA0A00??1221AAA0AAAA1A?AAAAAAAA

AAAAAAAAAAAAAAAAAAAAAAAAAAAAAAAAAAA0AAA12A?AAAAA0A2

AAAA111

Metaxytherium_medium____@sirenia

AAA2AA0A1A11A100AAAA0A00AAAAAAA1AAAA100AAA00A0AAAAA1AA

AAAAAAAAAA1A1AAA2A002A11AAA01A020A0001A1AAA1A0A111A1AAAA

AAAAA1AA1AA431A21111AAAAAAA0A00001221AAA0AAAA1A?AAAAAAA

AAAAAAAAAAAAAAAAAAAAAAAAAAAAAAAAAAA0AAA12A?AAAAA0A

2AAAA1??

Metaxytherium_serresii____@sirenia

AAA2AA0A1A11A100AAAA0A00AAAAAAA1AAAA100AAA00A0AAAAA1AA

AAAAAAAAAA1A1AAA2A002A11AAA01A020A0001A1AAA1A1A111A1AAAA

AAAAA1AA1AA431A21111AAAAAAA0A01001221AAA0AAAA1A?AAAAAAA

AAAAAAAAAAAAAAAAAAAAAAAAAAAAAAAAAAA0AAA12A?AAAAA0A

2AAAA111

Miosiren_kocki____@sirenia

AAA1AA0A1A10A101AAAA0A?1AAAAAAA0AAAA101AAA00A0AAAAA1AA

AAAAAAAAAA1A0AAA1A022A10AAA02A020A0001A0AAA1A0A111A1AAAA

AAAAA1AA1AA???A?????AAAAAAA0A02001211AAA0AAAA1A1AAAAAAAA

AAAAAAAAAAAAAAAAAAAAAAAAAAAAAAAAAAA0AAA?2A?AAAAA?A2A

AAA???

Nanosiren_garciae____@sirenia

AAA2AA?A1A10A?10AAAA0A0?AAAAAAA1AAAA?10AAA1?A1AAAAA1AAA

AAAAAAAAA1A1AAA1A102A13AAA01A01?A001?A?AAA?A0A121A1AAAAA

AAAA2AA1AA?31A2?11?AAAAAAA?A00??1221AAA0AAAA1A?AAAAAAAAA

AAAAAAAAAAAAAAAAAAAAAAAAAAAAAAAAAA?AAA??A?AAAAA0A?AA

AA?01

Nanosiren_sanchezi____@sirenia

AAA2AA0A1A10A?10AAAA0A00AAAAAAA1AAAA?10AAA1?A1AAAAA1AAA

AAAAAAAAA1A1AAA1A102A13AAA01A01?A001?A?AAA?A0A???A1AAAAA

AAAA2AA1AA???A?????AAAAAAA0A00001221AAA0AAAA1A?AAAAAAAAA

AAAAAAAAAAAAAAAAAAAAAAAAAAAAAAAAAA?AAA??A?AAAAA?A?AA

AA???

Pezosiren_portelli____@sirenia

AAA1AA0A1A11A?0?AAAA?A??AAAAAAA0AAAA00?AAA00A0AAAAA0AAA

AAAAAAAAA0A0AAA0A000A00AAA01A0??A????A?AAA?A0A0?0A0AAAAAA

AAA1AA1AA110A10000AAAAAAA0A01000100AAA0AAAA1A0AAAAAAAAA

AAAAAAAAAAAAAAAAAAAAAAAAAAAAAAAAA0AAA01A0AAAAA0A0A

AAA011

Phosphatherium_escuillei____@sirenia

AAA0AA0A0A01A00?AAAA0A?1AAAAAAA0AAAA00?AAA00A0AAAAA0AAA

AAAAAAAAA?A0AAA0A0??A00AAA?0A?00A000?A0AAA0A0A000A0AAAAA

AAAA0AA0AA?00A00?00AAAAAAA0A00000001AAA0AAAA0A0AAAAAAAA

AAAAAAAAAAAAAAAAAAAAAAAAAAAAAAAAAA?AAA??A?AAAAA?A?A

AAA???

Prorastomus_sirenoides____@proboscidea

AAA1AA0A1A11A000AAAA0A?0AAAAAAA0AAAA000AAA00A0AAAAA?AAA

AAAAAAAAA0A0AAA0A001A00AAA00A000A00?0A0AAA0A0A000A0AAAAA

AAAA0AA1AA100A00000AAAAAAA?A0?0?0000AAA0AAAA1A0AAAAAAAAA

AAAAAAAAAAAAAAAAAAAAAAAAAAAAAAAAAA?AAA??A?AAAAA?A?AA

AA???

Protosiren_fraasi____@sirenia

AAA1AA0A1A10A000AAAA0A?1AAAAAAA0AAAA000AAA00A0AAAAA1AA

AAAAAAAAAA0A0AAA0A011A00AAA10A000A00??A0AAA0A0A001A1AAAA

AAAAA1AA1AA210A??100AAAAAAA0A00000100AAA0AAAA1A1AAAAAAA

AAAAAAAAAAAAAAAAAAAAAAAAAAAAAAAAAAA?AAA??A?AAAAA?A?

AAAA???

Protosiren_smithae____@sirenia

AAA1AA0A1A10A000AAAA?A01AAAAAAA1AAAA000AAA00A0AAAAA1AA

AAAAAAAAAA0A1AAA1A011A00AAA10A000A00??A0AAA0A0A001A1AAAA

AAAAA?AA1AA210A11101AAAAAAA?A020?0100AAA0AAAA1A1AAAAAAAA

AAAAAAAAAAAAAAAAAAAAAAAAAAAAAAAAAA0AAA02A?AAAAA0A0

AAAA000

Rytiodus_capgrandi____@sirenia

AAA2AA2A1A??A?0?AAAA?A??AAAAAAA1AAAA31?AAA20A0AAAAA1AAA

AAAAAAAAA?A0AAA1A?02A?1AAA0?A020A?0??A1AAA?A?A?1?A1AAAAAA

AAA1AA1AA???A?????AAAAAAA1A0231???1AAA0AAAA1A?AAAAAAAAAA

AAAAAAAAAAAAAAAAAAAAAAAAAAAAAAAA?AAA??A?AAAAA?A?AAA

A???

Rytiodus_heali____@sirenia

AAA2AA2A1A10A200AAAA0A00AAAAAAA1AAAA310AAA20A0AAAAA1AA

AAAAAAAAAA0A1AAA1A00?A01AAA0?A020A1011A1AAA2A0A111A1AAAA

AAAAA1AA1AA?31A??11?AAAAAAA1A02311221AAA0AAAA1A?AAAAAAAA

AAAAAAAAAAAAAAAAAAAAAAAAAAAAAAAAAA?AAA??A?AAAAA?A?A

AAA???

Trichechus_inunguis____@sirenia

AAA1AA0A1A11A101AAAA0A01AAAAAAA1AAAA000AAA00A0AAAAA1AA

AAAAAAAAAA0A0AAA3A112A10AAA12A000A00?3A0AAA1A1A111A1AAAA

AAAAA2AA1AA320A22100AAAAAAA?A10??1221AAA1AAAA1A?AAAAAAAA

AAAAAAAAAAAAAAAAAAAAAAAAAAAAAAAAAA1AAA23A0AAAAA1A2

AAAA112

Trichechus_manatus____@sirenia

AAA1AA0A1A11A101AAAA0A01AAAAAAA1AAAA000AAA10A0AAAAA1AA

AAAAAAAAAA0A0AAA1A112A10AAA12A100A00?3A0AAA1A1A111A1AAAA

AAAAA2AA1AA320A22101AAAAAAA?A10??1221AAA1AAAA1A?AAAAAAAA

AAAAAAAAAAAAAAAAAAAAAAAAAAAAAAAAAA1AAA23A0AAAAA2A2

AAAA112

Trichechus_senegalensis____@sirenia

AAA1AA0A1A11A101AAAA0A01AAAAAAA1AAAA000AAA10A0AAAAA1AA

AAAAAAAAAA0A0AAA1A012A10AAA12A100A00?3A0AAA1A1A111A1AAAA

AAAAA2AA1AA320A22101AAAAAAA?A10??1221AAA1AAAA1A?AAAAAAAA

AAAAAAAAAAAAAAAAAAAAAAAAAAAAAAAAAA1AAA23A0AAAAA2A2

AAAA112

Metaxytherium_albifontanum____@sirenia

AAA2AA0A1A10A100AAAA0A00AAAAAAA1AAAA100AAA00A0AAAAA1AA

AAAAAAAAAA1A1AAA2A002A11AAA01A010A0001A1AAA1A0A111A1AAAA

AAAAA?AA1AA431A2?111AAAAAAA0A00001221AAA0AAAA1A?AAAAAAAA

AAAAAAAAAAAAAAAAAAAAAAAAAAAAAAAAAA0AAA12A1AAAAA0A2

AAAA111

Priscosiren_atlantica____@sirenia

AAA2AA0A1A10A100AAAA0A00AAAAAAA1AAAA000AAA00A0AAAAA1AA

AAAAAAAAAA0A0AAA0A002A11AAA01A020A0001A1AAA1A0A111A1AAAA

AAAAA1AA1AA431A21101AAAAAAA?A000?1221AAA0AAAA1A?AAAAAAAA

AAAAAAAAAAAAAAAAAAAAAAAAAAAAAAAAA?AAA12A?AAAAA?A?A

AAA???

Xenosiren_yucateca____@sirenia

AAA?AA2A1A?0A?21AAAA1A0?AAAAAAA1AAAA310AAA20A1AAAAA?AAA

AAAAAAAAA?A?AAA?A???A?1AAA0?A01?A111?A?AAA?A0A???A?AAAAAA

AAA?AA?AA???A?????AAAAAAA1A02?1???1AAA0AAAA1A?AAAAAAAAAA

AAAAAAAAAAAAAAAAAAAAAAAAAAAAAAAA?AAA??A?AAAAA?A?AAA

A???

Sobrarbesiren____@sirenia

AAA2AA0A1A11A000AAAA0A00AAAAAAA0AAAA00?AAA00A0AAAAA1AA

AAAAAAAAAA?A0AAA1A?02A11AAA00A0?0A00?0A0AAA?A0A011A1AAAA

AAAAA?AA?AA???A?????AAAAAAA0A00100100AAA0AAAA1A1AAAAAAAA

AAAAAAAAAAAAAAAAAAAAAAAAAAAAAAAAA?AAA?1A?AAAAA0A0A

AAA001

Eotheroides_lambondrano____@sirenia

AAA2AA1A1A1?A11?AAAA0A00AAAAAAA0AAAA00?AAA00A0AAAAA1AAA

AAAAAAAAA0A?AAA?A???A?1AAA0?A010A?101A1AAA?A0A???A1AAAAAA

AAA?AA?AA???A?????AAAAAAA?A0???????AAA?AAAA?A?AAAAAAAAAAA

AAAAAAAAAAAAAAAAAAAAAAAAAAAAAAA?AAA??A?AAAAA?A?AAAA

???

Eotheroides_sandersi____@sirenia

AAA1AA[01]A1A1?A11?AAAA?A?0AAAAAAA0AAAA?0?AAA00A0AAAAA1A

AAAAAAAAAAA0A0AAA1A001A11AAA0?A000A?101A1AAA0A0A1??A1AAA

AAAAAA2AA?AA110A????0AAAAAAA?A0???0101AAA0AAAA1A1AAAAAAA

AAAAAAAAAAAAAAAAAAAAAAAAAAAAAAAAAAAA0AAA02A?AAAAA0A

1AAAA?1[01]

Eotheroides_clavigerum____@sirenia

AAA1AA[01]A1A??A11?AAAA?A?0AAAAAAA0AAAA00?AAA00A0AAAAA1A

AAAAAAAAAAA0A0AAA?A0??A11AAA0?A000A??01A?AAA?A0A1??A1AAAA

AAAAA?AA?AA110A21?00AAAAAAA0A000?0101AAA0AAAA1A1AAAAAAAA

AAAAAAAAAAAAAAAAAAAAAAAAAAAAAAAAAAA0AAA?2A?AAAAA0A2A

AAA?1[01]

Prototherium_veronense____@sirenia

AAA1AA0A1A11A100AAAA?A10AAAAAAA0AAAA000AAA00A0AAAAA1AA

AAAAAAAAAA1A0AAA[01]A???A11AAA01A000A001?A0AAA1A?A1[12]1A1A

AAAAAAAA?AA?AA?20A10100AAAAAAA0A00100101AAA0AAAA1A1AAAAA

AAAAAAAAAAAAAAAAAAAAAAAAAAAAAAAAAAAAA?AAA??A?AAAAA

0A?AAAA?0?

Prototherium_intermedium____@sirenia

AAA2AA0A1A10A?00AAAA?A10AAAAAAA0AAAA000AAA00A0AAAAA1AAA

AAAAAAAAA0A0AAA[01]A???A11AAA0?A000A000?A?AAA?A0A11?A?AAAA

AAAAA?AA?AA?10A21100AAAAAAA0A01??010?AAA0AAAA1A1AAAAAAAA

AAAAAAAAAAAAAAAAAAAAAAAAAAAAAAAAAAA?AAA??A?AAAAA0A?A

AAA?0?

Prototherium_ausetanum____@sirenia

AAA1AA0A1A10A??0AAAA?A00AAAAAAA0AAAA00?AAA00A0AAAAA1AAA

AAAAAAAAA1A0AAA1A?0?A11AAA0?A00?A0000A?AAA0A0A111A1AAAAA

AAAA?AA?AA???A?????AAAAAAA?A?????101AAA0AAAA0A1AAAAAAAAAA

AAAAAAAAAAAAAAAAAAAAAAAAAAAAAAAAA?AAA??A?AAAAA?A?AAA

A???

Libysiren_sickenbergi____@sirenia

AAA1AA0A1A10A100AAAA0A?1AAAAAAA0AAAA00?AAA00A0AAAAA1AAA

AAAAAAAAA0A0AAA1A012A01AAA10A000A00?1A2AAA?A?A00?A1AAAAA

AAAA?AA1AA210A?1100AAAAAAA?A00??0100AAA0AAAA1A1AAAAAAAAA

AAAAAAAAAAAAAAAAAAAAAAAAAAAAAAAAAA?AAA??A?AAAAA?A?AA

AA???

;

Ccode

-[/1 0 -[/1 1 -[/1 2 +[/1 3 -[/1 4

-[/1 5 -[/1 6 -[/1 7 -[/1 8 -[/1 9

-[/1 10 -[/1 11 -[/1 12 +[/1 13 +[/1 14

-[/1 15 -[/1 16 -[/1 17 -[/1 18 -[/1 19

-[/1 20 -[/1 21 ([/1 22 -[/1 23 -[/1 24

-[/1 25 -[/1 26 -[/1 27 -[/1 28 -[/1 29

-[/1 30 -[/1 31 -[/1 32 -[/1 33 -[/1 34

-[/1 35 -[/1 36 -[/1 37 -[/1 38 -[/1 39

-[/1 40 -[/1 41 +[/1 42 -[/1 43 -[/1 44

-[/1 45 -[/1 46 -[/1 47 -[/1 48 -[/1 49

-[/1 50	-[/1 51	-[/1 52	-[/1 53	-[/1 54
-[/1 55	-[/1 56	-[/1 57	-[/1 58	-[/1 59
-[/1 60	-[/1 61	-[/1 62	-[/1 63	-[/1 64
-[/1 65	-[/1 66	-[/1 67	-[/1 68	-[/1 69
-[/1 70	-[/1 71	-[/1 72	-[/1 73	-[/1 74
-[/1 75	-[/1 76	([/1 77	-[/1 78	-[/1 79
-[/1 80	-[/1 81	-[/1 82	-[/1 83	-[/1 84
+[/1 85	-[/1 86	-[/1 87	-[/1 88	-[/1 89
-[/1 90	+[/1 91	-[/1 92	-[/1 93	-[/1 94
-[/1 95	-[/1 96	+[/1 97	-[/1 98	-[/1 99
-[/1 100	-[/1 101	-[/1 102	-[/1 103	-[/1 104
-[/1 105	-[/1 106	-[/1 107	-[/1 108	-[/1 109
-[/1 110	-[/1 111	-[/1 112	-[/1 113	-[/1 114
+[/1 115	-[/1 116	-[/1 117	-[/1 118	-[/1 119
-[/1 120	-[/1 121	([/1 122	-[/1 123	-[/1 124
+[/1 125	+[/1 126	+[/1 127	-[/1 128	-[/1 129
-[/1 130	-[/1 131	-[/1 132	-[/1 133	-[/1 134
-[/1 135	-[/1 136	-[/1 137	-[/1 138	-[/1 139
-[/1 140	-[/1 141	-[/1 142	-[/1 143	+[/1 144
+[/1 145	-[/1 146	-[/1 147	-[/1 148	-[/1 149

-[/1 150 -[/1 151 -[/1 152 -[/1 153 -[/1 154

+[/1 155 -[/1 156 +[/1 157 -[/1 158 -[/1 159

-[/1 160 -[/1 161 -[/1 162 -[/1 163 -[/1 164

-[/1 165 -[/1 166 -[/1 167 -[/1 168 -[/1 169

-[/1 170 -[/1 171 -[/1 172 -[/1 173 -[/1 174

-[/1 175 -[/1 176 -[/1 177 -[/1 178 -[/1 179

-[/1 180 -[/1 181 -[/1 182 -[/1 183 -[/1 184

-[/1 185 -[/1 186 -[/1 187 -[/1 188 -[/1 189

-[/1 190 -[/1 191 -[/1 192 -[/1 193 -[/1 194

-[/1 195 -[/1 196 -[/1 197 -[/1 198 -[/1 199

-[/1 200 -[/1 201 -[/1 202 -[/1 203 +[/1 204

+[/1 205 -[/1 206 -[/1 207 -[/1 208 -[/1 209

-[/1 210 -[/1 211 -[/1 212 +[/1 213 -[/1 214

+[/1 215 -[/1 216 -[/1 217 -[/1 218 -[/1 219

-[/1 220 ;

cost 22 = 0>1 1 0>2 1 0>3 1 0>4 1 0>5 1 0>6 1 0>7 1 0>8 1 0>9 1 0>10 1 1>0 1 1>2 2 1>3 1 1>4 1 1>5 1 1>6 1 1>7 1 1>8 1 1>9 1 1>10 1 2>0 1 2>1 2 2>3 1 2>4 1 2>5 1 2>6 1 2>7 1 2>8 1 2>9 1 2>10 1 3>0 1 3>1 1 3>2 1 3>4 1 3>5 1 3>6 1 3>7 1 3>8 1 3>9 1 3>10 1 4>0 1 4>1 1 4>2 1 4>3 1 4>5 1 4>6 1 4>7 1 4>8 1 4>9 1 4>10 1 5>0 1 5>1 1 5>2 1 5>3 1 5>4 1 5>6 1 5>7 1 5>8 1 5>9 1 5>10 1 6>0 1 6>1 1 6>2 1 6>3 1 6>4 1 6>5 1 6>7 1 6>8 1 6>9 1 6>10 1 7>0 1 7>1 1 7>2 1 7>3 1 7>4 1 7>5 1 7>6 1 7>8 1 7>9 1

7>10 1 8>0 1 8>1 1 8>2 1 8>3 1 8>4 1 8>5 1 8>6 1 8>7 1 8>9 1 8>10 1 9>0 1 9>1 1 9>2 1 9>3 1 9>4 1 9>5 1 9>6 1 9>7 1 9>8 1 9>10 1 10>0 1 10>1 1 10>2 1 10>3 1 10>4 1 10>5 1 10>6 1 10>7 1 10>8 1 10>9 1;

cost 77 = 0>1 1 0>2 2 0>3 2 0>4 1 0>5 1 0>6 1 0>7 1 0>8 1 0>9 1 0>10 1 1>0 1 1>2 1 1>3 1 1>4 1 1>5 1 1>6 1 1>7 1 1>8 1 1>9 1 1>10 1 2>0 2 2>1 1 2>3 2 2>4 1 2>5 1 2>6 1 2>7 1 2>8 1 2>9 1 2>10 1 3>0 2 3>1 1 3>2 2 3>4 1 3>5 1 3>6 1 3>7 1 3>8 1 3>9 1 3>10 1 4>0 1 4>1 1 4>2 1 4>3 1 4>5 1 4>6 1 4>7 1 4>8 1 4>9 1 4>10 1 5>0 1 5>1 1 5>2 1 5>3 1 5>4 1 5>6 1 5>7 1 5>8 1 5>9 1 5>10 1 6>0 1 6>1 1 6>2 1 6>3 1 6>4 1 6>5 1 6>7 1 6>8 1 6>9 1 6>10 1 7>0 1 7>1 1 7>2 1 7>3 1 7>4 1 7>5 1 7>6 1 7>8 1 7>9 1 7>10 1 8>0 1 8>1 1 8>2 1 8>3 1 8>4 1 8>5 1 8>6 1 8>7 1 8>9 1 8>10 1 9>0 1 9>1 1 9>2 1 9>3 1 9>4 1 9>5 1 9>6 1 9>7 1 9>8 1 9>10 1 10>0 1 10>1 1 10>2 1 10>3 1 10>4 1 10>5 1 10>6 1 10>7 1 10>8 1 10>9 1;

cost 122 = 0>1 2 0>2 1 0>3 2 0>4 1 0>5 1 0>6 1 0>7 1 0>8 1 0>9 1 0>10 1 1>0 2 1>2 1 1>3 2 1>4 1 1>5 1 1>6 1 1>7 1 1>8 1 1>9 1 1>10 1 2>0 1 2>1 1 2>3 1 2>4 1 2>5 1 2>6 1 2>7 1 2>8 1 2>9 1 2>10 1 3>0 2 3>1 2 3>2 1 3>4 1 3>5 1 3>6 1 3>7 1 3>8 1 3>9 1 3>10 1 4>0 1 4>1 1 4>2 1 4>3 1 4>5 1 4>6 1 4>7 1 4>8 1 4>9 1 4>10 1 5>0 1 5>1 1 5>2 1 5>3 1 5>4 1 5>6 1 5>7 1 5>8 1 5>9 1 5>10 1 6>0 1 6>1 1 6>2 1 6>3 1 6>4 1 6>5 1 6>7 1 6>8 1 6>9 1 6>10 1 7>0 1 7>1 1 7>2 1 7>3 1 7>4 1 7>5 1 7>6 1 7>8 1 7>9 1 7>10 1 8>0 1 8>1 1 8>2 1 8>3 1 8>4 1 8>5 1 8>6 1 8>7 1 8>9 1 8>10 1 9>0 1 9>1 1 9>2 1 9>3 1 9>4 1 9>5 1 9>6 1 9>7 1 9>8 1 9>10 1 10>0 1 10>1 1 10>2 1 10>3 1 10>4 1 10>5 1 10>6 1 10>7 1 10>8 1 10>9 1;

;

Ancstates

-0 -1 -2 -3 -4 -5 -6 -7 -8 -9

-10	-11	-12	-13	-14	-15	-16	-17	-18	-19
-20	-21	-22	-23	-24	-25	-26	-27	-28	-29
-30	-31	-32	-33	-34	-35	-36	-37	-38	-39
-40	-41	-42	-43	-44	-45	-46	-47	-48	-49
-50	-51	-52	-53	-54	-55	-56	-57	-58	-59
-60	-61	-62	-63	-64	-65	-66	-67	-68	-69
-70	-71	-72	-73	-74	-75	-76	-77	-78	-79
-80	-81	-82	-83	-84	-85	-86	-87	-88	-89
-90	-91	-92	-93	-94	-95	-96	-97	-98	-99
-100	-101	-102	-103	-104	-105	-106	-107	-108	-109
-110	-111	-112	-113	-114	-115	-116	-117	-118	-119
-120	-121	-122	-123	-124	-125	-126	-127	-128	-129
-130	-131	-132	-133	-134	-135	-136	-137	-138	-139
-140	-141	-142	-143	-144	-145	-146	-147	-148	-149
-150	-151	-152	-153	-154	-155	-156	-157	-158	-159
-160	-161	-162	-163	-164	-165	-166	-167	-168	-169
-170	-171	-172	-173	-174	-175	-176	-177	-178	-179
-180	-181	-182	-183	-184	-185	-186	-187	-188	-189
-190	-191	-192	-193	-194	-195	-196	-197	-198	-199
-200	-201	-202	-203	-204	-205	-206	-207	-208	-209

-210 -211 -212 -213 -214 -215 -216 -217 -218 -219

-220 ;

smatrix =0 (StepMatrix22)

0/1 1 0/2 1 0/3 1 0/4 1 0/5 1 0/6 1

0/7 1 0/8 1 0/9 1 0/A 1 1/2 2 1/3 1

1/4 1 1/5 1 1/6 1 1/7 1 1/8 1 1/9 1

1/A 1 2/3 1 2/4 1 2/5 1 2/6 1 2/7 1

2/8 1 2/9 1 2/A 1 3/4 1 3/5 1 3/6 1

3/7 1 3/8 1 3/9 1 3/A 1 4/5 1 4/6 1

4/7 1 4/8 1 4/9 1 4/A 1 5/6 1 5/7 1

5/8 1 5/9 1 5/A 1 6/7 1 6/8 1 6/9 1

6/A 1 7/8 1 7/9 1 7/A 1 8/9 1 8/A 1

9/A 1 ;

smatrix =1 (StepMatrix77)

0/1 1 0/2 2 0/3 2 0/4 1 0/5 1 0/6 1

0/7 1 0/8 1 0/9 1 0/A 1 1/2 1 1/3 1

1/4 1 1/5 1 1/6 1 1/7 1 1/8 1 1/9 1

1/A 1 2/3 2 2/4 1 2/5 1 2/6 1 2/7 1

2/8 1 2/9 1 2/A 1 3/4 1 3/5 1 3/6 1

3/7 1 3/8 1 3/9 1 3/A 1 4/5 1 4/6 1

4/7 1 4/8 1 4/9 1 4/A 1 5/6 1 5/7 1

5/8 1 5/9 1 5/A 1 6/7 1 6/8 1 6/9 1

6/A 1 7/8 1 7/9 1 7/A 1 8/9 1 8/A 1

9/A 1 ;

smatrix =2 (StepMatrix122)

0/1 2 0/2 1 0/3 2 0/4 1 0/5 1 0/6 1

0/7 1 0/8 1 0/9 1 0/A 1 1/2 1 1/3 2

1/4 1 1/5 1 1/6 1 1/7 1 1/8 1 1/9 1

1/A 1 2/3 1 2/4 1 2/5 1 2/6 1 2/7 1

2/8 1 2/9 1 2/A 1 3/4 1 3/5 1 3/6 1

3/7 1 3/8 1 3/9 1 3/A 1 4/5 1 4/6 1

4/7 1 4/8 1 4/9 1 4/A 1 5/6 1 5/7 1

5/8 1 5/9 1 5/A 1 6/7 1 6/8 1 6/9 1

6/A 1 7/8 1 7/9 1 7/A 1 8/9 1 8/A 1

9/A 1 ;

xgroup

;

agroup

;

cnames

{3 Rostrum;

{6 Nasal_processes_of_premaxillae;

{8 External_nares;

{10 Premaxillary_symphysis;

{11 Level_of_zygomatic-orbital_bridge_of_maxilla;

{13 'Infraorbital_foramen';

{14 Length_of_zygomatic-orbital_bridge_of_Maxilla;

{16 Palate;

{20 Infraorbital_canal;

{22 Edges_of_zygomatic-orbital_bridge_of_maxilla;

{23 Anterior_palate;

{31 Nasals;

{36 Supraorbital_process_of_frontal;

{37 Nasal_incisure_at_posterior_end_of_mesorostral_fossa;

{38 Frontal,_lamina_orbitalis;

{42 Frontal_roof_shape;

{44 Supraorbital_process_of_frontal;

{45 Frontal_roof_bosses;

{51 Sagittal_crest;

{64 Supraoccipital;

{66 Union_of_exoccipitals;

{70 Exoccipital,_dorsolateral_border;

{72 'Hypoglossal_foramen';

{73 'Postympanic_process_of_squamosal';

{74 Squamosal,_sigmoid_ridge;

{76 Cranial_portion_of_squamosal;

{77 Processus_retroversus_of_squamosal;

{81 'Outline_of_zygomatic_process_of_squamosal';

{82 'External_auditory_meatus_of_squamosal';

{84 'Zygomatic_process_of_squamosal';

{85 'Ventral_extremity_of_jugal';

{87 'Preorbital_process_of_jugal contact with;

{88 'Preorbital_process_of_jugal';

{89 Zygomatic_process_of_jugal;

{90 'Ventral_rim_of_orbit';

{91 Lacrimal_foramen;

{93 Lacrimal-premaxilla_contact;

{97 Posterior_border_of_palatine;

{99 Anterior_border_of_palatine;

{101 'Alisphenoid_canal';

{102 'Pterygoid_fossa';

{103 'Foramen_ovale';

{105 Sphenopalatine_region;

{115 Periotic;

{118 Mastoid_portion_of_periotic;

{121 Mandibular_symphysis;

{122 Ventral_border_of_horizontal_mandibular_ramus;

{123 'Accesory_mental_foramina';

{125 'Posterior_border_of_mandible';

{126 Anterior_border_of_coronoid_process;

{127 Mandibular_dental_capsule;

{128 Horizontal_ramus_of_mandible;

{129 Ventral_border_of_horizontal_ramus_of_mandible;

{137 First_upper_incisor;

{139 First_upper_incisor;

{140 Depth_of_I1_alveolus;

{141 Cross_section_of_I1_crown;

{142 First_upper_incisor;

{143 Second_and_third_upper_incisors,_first_through_third_lower_incisors;

{144 Canines;

{145 Premolars_1-4;

{146 Permanent_premolar_5;

{150 Supernumerary_molars;

{155 Postcanine_dental_formulaCharacter_64;

{157 Permanent_premolars;

{200 Cervical_vertebrae;

{204 Lumbar_vertebrae;

{205 Sacrum;

{207 'Posterior_caudal_vertebrae';

{213 Bicipital_groove_of_humerus;

{215 Pubis;

{220 Tibia;

{221 Humerus;

{222 Deltoid_process_of_humerus;

;

taxcode

+0 +1 +2 +3 +4 +5 +6 +7

+8 +9 +10 +11 +12 +13 +14 +15

+16 +17 +18 +19 +20 +21 +22 +23

+24 +25 +26 +27 +28 +29 +30 +31

+32 +33 +34 +35 +36 +37 +38 +39

+40 +41 +42 +43 +44 +45 +46 +47

+48 +49

;

blocks 0;

Outgroup 13;

Outgroup[proboscidea;

Hold 50000;

proc/;

References

38. Dreyer, T., Corregidor, J., Arbues, P. & Puigdefabregas, C. Architecture of the tectonically influenced Sobrarbe deltaic complex in the Ainsa Basin, northern Spain. *Sediment. Geol.* **127,** 127–169 (1999).

39. Abel, O. Die eocänen Sirenen der Mittelmeerregion. Erster Teil. Der Schädel von *Eotherium aegyptiacum*. *Palaeontographica* **59,** 289–360 (1913).

40. Bizzotto, B. La struttura cranica di *Prototherium intermedium* (Mammalia: Sirenia) del l´Eocene superiore veneto. Nuovi contributi alla sua anatomia e sistematica. *Lav. Soc. Veneziana Sci. Nat.* **30,** 107–125 (2005).

41. Domning, D. P. Sirenian evolution in the North Pacific Ocean. *University of California Publications in Geological Sciences* **118**, 1-179 (1978).
